# Phytoremediation Potential of Native Plant Species in Mine Soils Polluted by Metal(loid)s and Rare Earth Elements

**DOI:** 10.3390/plants12061219

**Published:** 2023-03-07

**Authors:** Mitra Azizi, Angel Faz, Raul Zornoza, Silvia Martinez-Martinez, Jose A. Acosta

**Affiliations:** Sustainable Use, Management and Reclamation of Soil and Water Research Group, Universidad Politécnica de Cartagena, Paseo Alfonso XIII 48, 30203 Cartagena, Spain

**Keywords:** phytoextraction, phytostabilization, mining activity, bioacumulation

## Abstract

Mining activity has an adverse impact on the surrounding ecosystem, especially via the release of potentially toxic elements (PTEs); therefore, there is an urgent need to develop efficient technologies to remediate these ecosystems, especially soils. Phytoremediation can be potentially used to remediate contaminated areas by potentially toxic elements. However, in soils affected by polymetallic contamination, including metals, metalloids, and rare earth elements (REEs), it is necessary to evaluate the behavior of these toxic elements in the soil-plant system, which will allow the selection of the most appropriate native plants with phytoremediation potential to be used in phytoremediation programs. This study was conducted to evaluate the level of contamination of 29 metal(loid)s and REEs in two natural soils and four native plant species (*Salsola oppositifolia*, *Stipa tenacissima*, *Piptatherum miliaceum*, and *Artemisia herba-alba*) growing in the vicinity of a Pb-(Ag)-Zn mine and asses their phytoextraction and phytostabilization potential. The results indicated that very high soil contamination was found for Zn, Fe, Al, Pb, Cd, As, Se, and Th, considerable to moderate contamination for Cu, Sb, Cs, Ge Ni, Cr, and Co, and low contamination for Rb, V, Sr, Zr, Sn, Y, Bi and U in the study area, dependent of sampling place. Available fraction of PTEs and REEs in comparison to total concentration showed a wide range from 0% for Sn to more than 10% for Pb, Cd, and Mn. Soil properties such as pH, electrical conductivity, and clay content affect the total, available, and water-soluble concentrations of different PTEs and REEs. The results obtained from plant analysis showed that the concentration of PTEs in shoots could be at a toxicity level (Zn, Pb, and Cr), lower than toxic but more than sufficient or natural concentration accepted in plants (Cd, Ni, and Cu) or at an acceptable level (e.g., V, As, Co, and Mn). Accumulation of PTEs and REEs in plants and the translocation from root to shoot varied between plant species and sampling soils. *A. herba-alba* is the least efficient plant in the phytoremediation process; *P. miliaceum* was a good candidate for phytostabilization of Pb, Cd, Cu, V, and As, and *S. oppositifolia* for phytoextraction of Zn, Cd, Mn, and Mo. All plant species except *A. herba-alba* could be potential candidates for phytostabilization of REEs, while none of the plant species has the potential to be used in the phytoextraction of REEs.

## 1. Introduction

Since the industrial revolution, mining activities have been one of the most important sources of anthropogenic contamination in several soils around the world, particularly in regions with a long history of activity. One of the main concerns regarding mining operations is an inappropriate or uncontrolled abundance of numerous tailing deposits that contain a high content of potentially toxic elements (PTEs), during exploitation for metal(loid) extraction or after the closure of the activity, without any remediation treatments. These PTEs include metals (e.g., Zn, Pb, Cd, Cu, U, and Th), metalloids (e.g., Ge, Sb, As, and Bi), and rare earth elements (REEs) (e.g., Pr, Y, and La) which, especially in high concentrations, have seriously affected the physiological and biochemical processes in plants, animals, humans, as well as soil microorganisms [1,2,3,4]. In addition to the pollution in abandoned mine tailings ponds, previous studies have demonstrated that these places have a great adverse impact on the surrounding ecosystem by the transportation of PTEs through wind, surface water (including acid mine drainage, i.e., AMD), and groundwater seepage. Therefore, the distribution of PTEs throughout these districts could be affected by natural factors such as terrain (elevation and slope), wind direction, rainfall, and the distances from rivers as well as mine tailings PTE contents [5,6,7,8,9]. Additionally, the physic-chemical properties of soil, such as pH, electrical conductivity, etc., affect the mobility of PTEs [10,11]. In fact, chemical reactions between these elements and solid components of soil determine their bioavailability and solubility; Dimirkow et al. [11] concluded that Cd adsorption by goethite and clinoptilolite increases with the increase in pH and with the decrease in electrolyte concentration. In addition, sometimes, the lands around abandoned mines are used for agriculture, as well as for children’s parks or as tourist areas [9,12,13]. These different land uses can result in various exposure pathways which enter the PTEs in the human body via the food chain or direct intake and can pose risks to human health and safety, especially to those residing in the vicinity of these areas [14].

Therefore, there is an urgent need to develop efficient and sustainable soil remediation programs to reduce the risk associated with the mobility, transportation, dispersion, and ecotoxicity of PTEs from abandoned mine tailings to surrounding environments. These programs should be based on studies of ecological and health risks assessment which allow the selection of the most appropriate measures to reduce these risks. Apart from several chemical/physical technologies such as excavation of contaminated material, landfilling, incineration, immobilization, stabilization/solidification, and chemical extraction, phytoremediation can provide a less invasive, low-cost phytotechnology that is an environmentally friendly, long-lasting, and aesthetic solution to rehabilitate these contaminated areas [15,16,17]. Phytoremediation is the process of application of green plants to remove or render PTE contaminants harmless. Among phytoremediation techniques, phytoextraction and phytostabilization are the most promising options for mine tailings and surrounding soil reclamation [17].

Phytostabilization, by establishing a plant cover on the surface of contaminated soils, aims to deactivate and immobilize the PTEs within a limited area through root accumulation or precipitation within the rhizosphere, thereby diminishing uptake and transport of PTEs and the chances of any biological interactions with humans or animals. In contrast to phytostabilization, phytoextraction relies on the ability of plants to absorb and accumulate soil PTEs in their shoots, so that they can later be harvested in order to remove metal(loid)s from the soil [16,18]. Moreover, it was suggested that the plants exhibiting greater BCF (bioconcentration factor) and less than 1 TF (translocation factor) can be used as potential candidates for the phytostabilization of metal(loid)s. Plants with BCF and TF both greater than one have the potential to be used for phytoextraction [16,17]. Several studies have shown the efficiency of phytoextraction in reducing the concentration of a toxic element in soil; p.e. Kolodziej et al. [19] found that giant miscanthus was able to recover 47% of Mo, 39% of Mn, and 35% of Fe from municipal sewage sludge. In addition, phytoextraction can be improved by the application of various amendments; Grzegordka et al. [20] concluded that the application of a soil improver increases by 85% the mass of *Miscanthus × giganteus* compared to the soil without additives, increasing the uptake of Al, Fe, Co, Pb, Mn, Ni, and Cd. Additionally, plants used for phytoremediation need to effectively tolerate high concentrations of metal(loid)s, grow rapidly, and their root systems must be vigorous and show proper adaptability against a specific area [16]. Native plants, which can be found in wide geographical locations, are of particular interest in this perspective, since these spontaneous plant species are genetically adapted to the contaminant and, as such, can remove or retain it, reducing its toxicity in soil [15,21]. They could belong to a group known as hyperaccumulators, which can tolerate, absorb, accumulate, and translocate high levels of metal(loid)s, or excluder plants that can maintain relatively low levels in their shoot while still containing large amounts of metal(loid)s in their roots [22]. Furthermore, numerous studies have shown that native plants that grow in contaminated areas are either more resistant through higher disposal efficiency or have more metal(loid) accumulation and resistance to stressful prevailing conditions than plants grown in non-contaminated areas. Many native plant species have been identified and selected as potential phytoremediation plants to extract or stabilize metal(loid)s in impacted mines, for example, *Salsola soda* [23], *Salsola*, *Eremopyrum*, *Aeluropus litoralis* [24], *Salsola oppositifolia Desf*, *Limonium delicatulum*, *Moricandia arvensis* (L.) DC [18], *Piptatherum caerulescens*, *Coris monspeliensis*, *Lobularia maritima* [25] and *Pinus halepensis*, and *Tetraclinisarticulata* [26]. However, there are very few studies that evaluate the potential of native plants to be used in phytoremediation programs in soils affected by polymetallic contamination, including heavy metals as well as metalloids and rare earth elements. In particular, few studies have evaluated the behavior of rare earth elements in contaminated soils and their uptake by native vegetation.

For hundreds of years, the Mazarron district (Murcia Region, southeast Spain) has been exposed to mining activity for Pb, Ag, and Zn extraction [27]. Although this site was abandoned in 1996, many tailing ponds remain in the area without any restoration [28]. The objective of this study was to test whether the two natural soils near this mining area are contaminated by metal(loid)s and REEs, and also to identify metal(loid) and REE accumulation patterns in native species present in soils and any relationships between the concentration of metals in plants and soils, in order to suggest suitable ones for use in phytoremediation (phytoextraction or phytostabilization) strategies.

## 2. Results and Discussion

### 2.1. Physic-Chemical Characteristics of Soils 

Table 1 summarizes the characteristic of soil profiles from Unit S1 and Unit S2. Profile 1 was taken in Unit S1, and it has been developed on dacites. Three horizons were identified: A1, A2, and C/R. The pH in all horizons is slightly alkaline, which favors the development of vegetation but can result in a deficiency of nutrients such as P or Fe that are not widely available or soluble at high pH. In this pH range, most of the PTEs are precipitated and have little availability, thus reducing the risk of transfer of pollutants by leaching, runoff, or through the trophic chain. All three horizons are non-saline. The content of OC and TN decreases in depth. CEC was less than 10 cmol^+^ kg^−1^, which represents a moderate capacity for nutrient adsorption [29,30]. The three horizons presented clay-sandy loam textural class, with a predominance of sand (61–66%). This profile is classified as Leptic Regosol [31]. 

Profile 2 was taken in the southwest of Unit S2. It has also been developed on dacites. Three horizons were identified: A, AC, and C/R (Table 1). The pH in all horizons was basic (8.04–8.18), which favors the development of vegetation; similarly to profile 1, all three horizons were non-saline. The content of OC and TN was higher in the surface horizon A, and decreased in depth, with relatively low values for forest soils, as a consequence of the degradation suffered by anthropic action. The horizons of this profile have a CEC lower than 10 cmol^+^ kg^−1^. The first two horizons of the profile have a clay-sandy loam textural class, with a predominance of sand (62–66%). The third horizon (C/R) presents a sandy loam texture, with a higher sand content (78%) than the upper horizons. Profile 2 is classified as Leptic calcisol [31]. Profile 3 was taken in the north of Unit S2, next to a *P. halepensis* pine forest. Like the previous profiles, this one has also been developed on dacites. Three horizons were identified: A, C1, and C2 (Table 1). The pH in all horizons was basic (7.92–8.26). All three horizons are non-saline. The OC and TN content and CEC pattern were similar to other profiles. The first two horizons of the profile presented a clay-sandy loam textural class, with a predominance of sand (59–68%). The third horizon (C2) had a clay-sandy texture, with a higher clay content (42%) than the upper horizons. Profile 3 is classified as Leptic Calcisol [31]. 

As shown in Table 1, the surface and subsurface soils in Unit S1 were slightly alkaline (7.86 and 7.97, respectively), which are lower than the pH from Unit S2 (8.13–8.27). The result from the previous study [27] showed that all the materials that made up the surface of mine ponds (sludge and gravimetric waste), which are near Unit S1 and Unit S2, showed a high acidity (3.86–3.15 and 4.89–3.15). A lower pH value of sludge near S1 [27] may result in lower pH values in the surface and subsurface soils of Unit S1 in comparison to Unit S2. In total, these pH conditions are suitable for plant growth, and it is also the key factor influencing the migration, adsorption, and precipitation of metal(loid)s and REEs in soil. 

The predominance of sand fraction in the natural soils (Table 1) and mining residues near them [27] probably resulted in wind and water erosion to mobilize these particles to the nearby environment and promote water infiltration [32]. These soil samples exhibited small EC values (0.13–0.19 dsm^−1^), indicating low transfer of salts from mine waste to natural soils. Previous studies reported a correlation between low pH and high EC, since the presence of high amounts of sulfur ions in mining residues led to an increase in the EC, while their oxidation caused pH reduction by the formation of sulfuric acid [33]. 

### 2.2. Metal(loid)s and REEs Concentrations in Soils

#### 2.2.1. Distribution of Total Concentration in Soils

Results showed that the highest concentrations of total metal(loid)s were mainly found for Fe, Al, Pb, Zn, and Mn in both Units (Table 2). The previous study [27] reported that mining waste materials in the vicinity of these natural soils are composed of high concentrations of Fe, Pb, and Zn that originated from jarosite, clinochlore, and halloysite minerals [28]. Therefore, the enrichment of these elements in these Units possibly came from mining waste materials. The mean concentrations of total Al and Zn in Unit S1 were 48,505 and 2436 mg kg^−1^, respectively, which were significantly higher than the corresponding values in Unit S2 (28,127 and 1187 mg kg^−1^, respectively) (Table 2). However, there were no significant differences in total concentrations of Al among mining wastes and different units [27] in the vicinity of these natural soils; the significant difference found in these natural units could be a result of different soil properties between these areas, such as pH, organic carbon, and carbonate (Table 1). Al is the most abundant metal in the Earth’s crust and part of the structural components of clays, while the excess amount of its concentration in soils is toxic to most plants [34]. It is well known that AMD could be responsible for Al release, especially at low pH ranges, and followed by precipitation happens when the pH values enhances to 4.0–5.5 [35]. Units S1 and S2, are located downstream of the open-cast mining, which leads to the release of Al from the current tailings mine which are disposed as open dump. 

Sphalerite (ZnS) is the main sulfide of the ore vein in sulfurous mining areas that may release Zn into the environment. The result from the previous study [27] demonstrated that the total Zn concentration in gravimetric waste, located near Unit S1, was significantly higher than other residues. Zhang et al. [5] showed that the amounts of Zn leaving a tailings pond at a copper mine with drainage water were 21.6% of the amounts released from oxidation in the oxidized zone. 

There is no significant difference in the contents of total Pb between Unit S1 and Unit S2 (Table 2). Gabarron et al. [28] concluded that the high concentration of Pb in natural soils could have happened as a result of Pb transfer from mining ponds, and parent material could be a secondary source of Pb pollution in agricultural and natural soil in the Mazarron district. On the other hand, the mean total Mn concentration in Unit S2 was higher than in Unit S1 (694 and 328 mg kg^−1^, respectively) (Table 2). Manganese forms many minerals such as todorokite, clinochlore, pyrolusite, and serandite and is highest in igneous rocks, gabbros, and basalts [36]. Furthermore, it is similar in size to Mg^2+^ and Fe^2+^, and their substitution in oxides and silicates may enhance the specific surface area that finally contributes to the flux control of many heavy metals such as Co, Ni, Cu, and Zn [37]. Moreover, Mn and Fe are involved in a wide spectrum of biogeochemical pathways such as mineral dissolution, microbial processes, the formation of a wide range of highly reactive solid phases (Fe and Mn oxy-hydroxides), and the biogeochemical cycles of other major elements (e.g., carbon, sulfur, and phosphorus) [38]. The result showed that the mean Fe concentration in Unit S1 was higher than in Unit S2 (58,318 and 38,408 mg kg^−1^, respectively) (Table 2). It was suggested that the mining districts of Mazarron are composed of high amounts of Fe-oxyhydroxides, Pb, Zn, and Fe sulfides, that are related to the parent materials [28,39].

The fifth most abundant metal in studied soils was obtained for Sr, which was 2.39 times higher in Unit S2 than the concentration found in Unit S1 (459 and 135 mg kg^−1^, respectively) (Table 2). The high amount of Sr in Unit S2 can be related to the high level of Sr in waste materials (495 mg kg^−1^) [27] that are located in the vicinity of the study area. Sr is a trace element with a common concentration between 260 and 730 mg kg^−1^ and belongs to the alkaline earth metals; it behaves similarly to Ca and Mg, and the main source of Sr pollution is associated with coal combustion and sulfur mining [40]. 

The mean As concentrations found in Unit S1 and Unit S2 soils were 131 and 149 mg kg^−1^, respectively (Table 2), which were similar to the amount found in agricultural soils from Mazarron (149 mg kg^−1^) in the research of Gabarron et al. [28]; however, there were no significant differences between these two Units. Gabarron et al. [28] suggested that the high concentrations of As can be associated with ferrous minerals common in the studied area as arsenopyrite (FeAsS). 

The result of our study showed that there is no significant difference between the total concentration of Cr, Ni, In, Sn, Sb, Bi, and Rb in Unit S1 and Unit S2 (the ranges are 110–86.5, 34.5–38.1, 0.87–1.40, 4.70–6.87, 3.06–4.20, 0.29–0.47, and 66.0–73.8 mg kg^−1^, respectively) (Table 2). Chromium is quite abundant in most soils, and chromite (FeCr_2_O_4_) and Crocoite (PbCrO_4_) are the most common Cr-minerals, which usually are associated with pyroxenes, amphibolites and micas and heavy metals such as Ni and Co [36,40]. According to Kabata-Pendias and Mukherjee [40], Ni primarily often forms sulfides and sulfarsenides together with Fe and Co, and is associated with several Fe minerals. After weathering, it coprecipitates with Fe and Mn oxides, and can also be associated with carbonates, phosphates, and silicates minerals [41]. 

Oppositely, higher total concentrations of Co, Cu, Zr, and Cs were observed in Unit S2 than in Unit S1 (11.7–4.06, 71.1–39.8, 15.8–4.96, 42.6–6.89 mg kg^−1^, respectively, which were 2.89, 1.78, 3.18, and 6.18 times higher) (Table 2). The acid character of the mining waste around those areas, which favors the mobility of these metals, leads to leakage of them to surrounding natural soils, that is, Units S2 and S1, where the sorption phenomena happened since the conditions such as greater pH and CEC are present [42]. 

It was also observed that the total concentration of V, Se, Y, Mo, Cd, La, Ce, Pr, Th, Ge, and U were significantly higher in Unit S1 in comparison to Unit S2 (83.9–72.3, 2.16–1.32, 23.8–14.9, 3.44–2.22, 14.1–6.67, 54.1–26.3, 118–61.8, 15.3–7.89, 36.7–17.3, 4.94–2.84, and 7.92–4.26 mg kg^−1^, respectively) (Table 2). The accumulation of these metal(loid)s and REEs in Unit S1 can be attributed to the higher concentration of waste materials located in the vicinity of this area [27]. The oxidation of sulfides within mining waste materials can accelerate the dissolution of REE-bearing minerals (e.g., carbonates, silicates, and phosphates) and enhance the leaching of REE and other associated contaminants such as uranium, thorium, and niobium [43,44,45]. Among different REEs, the highest concentration in both areas was Ce (Table 2). Pereira et al. [46] found the same result in a gold mining area in the Amazon. REEs are ingredients of several different minerals and can also be concentrated in phosphorites, being included in quite common minerals such as monazite ((CeLa)PO_4_), bastnasite ((Ce_F_)CO_3_), cheralite ((Ce, La, Y, Th)PO_4_), and xenotime (YPO_4_) [40], or as impurity elements spread in rock-forming minerals and rare metal minerals via isomorphic substitution. Such minerals are often mentioned as REE-containing minerals (e.g., apatite and fluorite) [47]. Mleczek et al. [44] mentioned that REE concentrations in the following few years may be related to a major new form of environmental pollutants, and this increase may also pose a hazard to both plant and human health. 

The evaluation of total metal(loid) and REE concentration distribution in depth (Table 2) showed that there is no significant difference between the surface (0–15 cm) and subsurface (15–30 cm), except for Co, Ni, La, Ce, Pr, Sn, Th, and Ge. However, there was no significant difference in total Ni by increasing depth in Unit S1; in Unit S2, a higher amount of Ni was accumulated in surface soil (1.46 times higher than in the subsurface) (Table 2). Moreover, in Unit S1, total Ce, Pr, and Th in the subsurface were 1.56, 1.46, and 1.87 times higher than surface soils, while they showed no differences in Unit S2 (Table 2). In addition, the total concentration of La and Ge increased with depth in Unit S1, and they were 1.58 and 1.16 times more than those found in the subsurface soils and adversely were 0.82 and 0.83 times lower in Unit S2. Therefore, Unit S1 favored accumulating these REEs at the subsurface and Unit S2 at the surface (Table 2). However, REE naturally tends to concentrate on the upper layers of a soil profile, as found in Ni, La, and Ge in Unit S2 [48]; they could occur at the depth of mine soils [49]. In general, REEs complexation in natural soils, which is known to enhance their mobility in soils, is mainly associated with clay minerals, organic matter, carbonates, and humic substances, or Fe (hydr)oxides and colloids [50,51], whereas phosphate complexation leads to decreased REE solubility [52].

#### 2.2.2. Contamination Factor 

**Figure 1:**
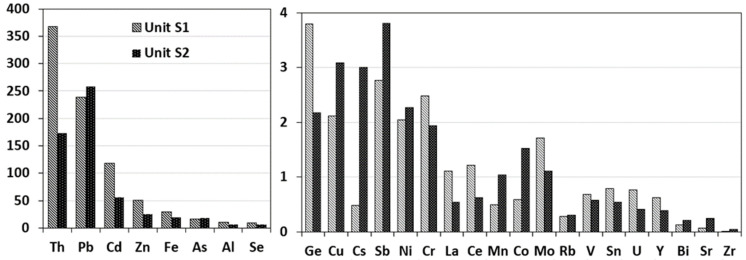
Contamination factor of metal(loid)s and REEs in the study area.

Based on the Contamination Factor (CF) values, varying degrees of contamination were observed for the different metal(loid)s and REEs studied (Figure 1). Very high contamination (CF ≥ 6) was found for Zn, Fe, Al, Pb, Cd, As, Se, and Th in both Unit S1 and Unit S2. Concentrations of Th, Pb, and Cd in Unit S1 and Unit S2 were 363–173, 239–258, and 117–55 times higher than background values [53], respectively, and exceed the proposed generic reference levels [53,54] (Figure). These high concentrations are mostly due to the mining activity that has contributed to concentrating and enriching of these pollutants and also to the geological substrate enriched with these elements, which finally bring high ecological risks to these soils [27]. Sahoo et al. [55], by the review of a total of 117 relevant reports from 19 different countries such as India, Iran, Slovakia, Brazil, China, and Morocco, reported that in soil plus overburden samples taken near mining sites, maximum CF values for Fe, Zn, Cd, Pb, and As were 10.5, 28.8, 320, 34, 754, respectively. Martinez-Carlos et al. [21] reported that As, Pb, Cd, and Zn exceed more than 10, 600, 40, and 80-fold the background level for natural soils in the study area located in the Cartagena-La Union mining district, Murcia Region (SE Spain), near the tailings ponds with a high concentration of Pb, Zn, Fe, Mn, or As. 

While considerable contamination (3 ≤ CF < 6) was found for Cu, Sb, and Cs in Unit S2 and Ge in Unit S1, which were 3.1, 3.8, 3, and 3.8 times higher than the regional background levels, moderate contamination (1 ≤ CF < 3) was found for Cu and Sb in Unit S1 and Ge in Unit S2 (Figure 1). In addition, Cs in Unit S1 showed low contamination (CF < 1) (Figure 1). Azizi et al. [27] reported that Cu, Sb, and Ge were more than 10, 8, and 4 times higher than the background level in waste materials of mine tailings in the vicinity of these natural soils, while Cs was within the background levels of the area. These results probably indicate that mine tailings could be responsible for the accumulation of these pollutants in the study area.

However, the total Co, Ni, and Cr content in the studied mining waste was within the background levels of these elements in the Region of Murcia [27]; CF values showed moderate contamination (1 ≤ CF < 3) for Ni, Cr, and Co in Unit S2 and Ni and Cr in Unit S1 (Figure 1). This probably happened as a result of leakage of these elements to surrounding soils or as dust deposition. In addition, moderate contamination (1 ≤ CF < 3) was found for Mo in both soils, Mn in Unit S2 and La and Ce in Unit S1 (Figure 1). Low contamination (CF < 1), which means the total metal concentration is below background levels, was found for Rb, V, Sr, Zr, Sn, Y, Bi, and U in both Unit S1 and Unit S2, Mn, Co, and Cs in Unit S1, and La and Ce in Unit S2 (Figure 1). Despite the total concentrations of Mn, Cs, Sn, Bi, and U being above the background levels of these elements in mining waste in this region [27], Rb, Sr, Zr, Y, La, V, Th, and Ce in mining ponds were below or only slightly exceeding the limits proposed by Ballesta et al. [56], which means the anthropic activities have a low impact on these soil metal concentrations in the study area, not presenting a significant risk and indicating a natural origin of these elements [27]. 

#### 2.2.3. Relation and Behavior among Metal(loid)s and REEs

In this study, total V, Sr, Zr, and Cs positively correlated with pH, while Zn, Al, Fe, Sn, Mo, Cd, Ge, La, Th, Pr, Ce, U, and Y negatively correlated with soil pH (Supplementary Material, Table S1). However, some studies did not show any correlation between the total metal content (Pb, Th, U, Zn, Cd, Rb, V, and Co) in the soil with pH [57]; others found a negative or positive correlation of total content of elements (Sb, As, and Cu) and (Cr, Ni, and As) in soils with pH, respectively [58,59]. Soils with a neutral and alkaline pH generally have high calcium carbonate content, and together with alkaline pH values, many dissolution and precipitation processes are controlled by pH, which can finally predict the retention or migration of metals in soil. Probably considering that the mining wastes are acid with a high PTE mobility, PTEs are spread from the contaminated landfills into the surrounding soils and to Unit areas, where the high pH favors their precipitation and accumulation. In addition, the wind deposition could contribute to this high level of PTEs in the Units evaluated.

Among all evaluated elements, only Sr had a significant positive correlation with EC, while V, Y, La, Ce, Pr, Th, Ge, Zn, and Al negatively correlated with EC (Supplementary Material, Table S1). Strontium may attribute in or near sedimentary rocks associated with beds or lenses of gypsum, anhydrite, and rock salt as well as in veins associated with limestone and dolomite, and dispersed in shales, marls, and sandstones [60], so the positive correlation with EC is possible. 

While most elements such as Ni, Mo, In, Ge, Zn, Fe, Se, and Sn are positively correlated with sand and negatively with clay, total Zr and Cs showed a negative correlation with sand fraction in the studied area (Supplementary Material, Table S1). Moreover, Bi positively correlated with sand and silt fraction, while it was negatively correlated with clay. In addition, Mn, Co, Cu, and Sr are also positively correlated with silt (Supplementary Material, Table S1). Adriano [61] reported that minerals present in silt fractions have elements such as Cu, Pb, Bi, Sn, and Fe in their composition. The accumulation of more alkali metals such as Cs, Rb, and K in the finer-than-sand fractions of the soils than in the sand fractions is a common phenomenon and is due to the dilution of the alkali metal-bearing phases with quartz [41]. In addition, the enrichment of coarser soil fraction by metals resulted in more distribution of these pollutants to nearby soils by wind and water erosion. 

The concentrations of several of the elements were positively correlated (Supplementary Material, Table S2). Significant positive correlations between REEs and metal(loid)s containing La, Ce, Pr, Th, Y, U, V, and Cd with Ge, Al, Zn, and Fe were found (Supplementary Material, Table S2). Moreover, a positive correlation between REEs and other metals such as Ce, Pr, Y, Th, Ni with Sn, and Se, and between REEs such as La, Ce, and Pr revealed that they had similar inputs or common geochemical characteristics [49]. Azizi et al. [27] indicated that Cr, Ni, La, Ce, Rb, Pr, Mo, U, Cd, Zn, V, and Co were associated with Al and/or Mn minerals as impurities. Like other studies, a positive correlation was found between Ni and Cr (Supplementary Material, Table S2), which can be concluded that transport, accumulation, and sources of Ni and Cr could be similar due to a high correlation between them [23]. Similar to tailing near the study area [27], a positive correlation was found between Bi and Pb in soils, which indicates the same origin (Supplementary Material, Table S2). 

#### 2.2.4. Available and Water-Soluble Metal(loid)s and Rees

In most cases, the total concentration of an element will not be available for immediate uptake by plants. The result showed that the concentration of available V, As, Sr, Sb, Rb, and Cs were higher in Unit S2 than in Unit S1, while Y and Cd were higher in Unit S1 than in Unit S2 (Table 3). The most available percentage of metal(loid)s and REEs evaluated were Pb, Cd, Mn, Cu, Sr, Co, Sb, Y, and Zn (14.45%, 16.53%, 10.04%, 6.98%, 5.30%, 4.09%, 3.37%, 3.24%, and 3.06% of total concentration, respectively). Available concentrations of Sn and Cs were almost equal to zero, and in the case of Cr, As, Th, and Rb were lower than 0.1 percent of the total content in the soils. The high amount of available fraction of contaminant in the soil will show whether this soil poses a possible risk of toxicity to some species of plants, soil fauna, or microorganisms [62]. Moreno-Jimenez et al. [62] showed that Cd and Mn were significantly more easily extractable than the other metals, while Cu showed low extractability and Fe was strongly retained in soils. Similar to our result, Loell et al. [63] showed that among different REEs, Yttrium was the most available element when the soil was extracted with ethylenediaminetetraacetic acid (EDTA). Availability is affected by many factors, including pH, redox status, macronutrient levels, available water content, and temperature [36]. Previous research showed that As tends to bind to a Fe oxide/hydroxide phase [64], Zn to CaCO_3,_ and Pb to Fe and Mn oxides and oxyhydroxides [65].

Correlation between soil properties and available concentration of elements showed that Cu, Mo, Cd, La, Bi, Rb, and Zn were negatively correlated with clay content (Supplementary Material, Table S1). Moreover, a significant negative correlation was found between available Y, Cd, La, Pr, Fe, and Zn and soil pH (Supplementary Material, Table S1). It can be stated that soils with a neutral and alkaline pH generally have high calcium carbonate content. Together with alkaline pH values, the presence of carbonates in the soil enhances the retention of metal(loid)s mainly as carbonate salts, as a consequence of the ionic exchange, which is the principal retention mechanism of metals [33].

Similar to available metal(loid)s and REEs, Unit S2 soils generally showed higher concentrations of water-soluble V, As, Sr, Sb, Rb, and Cs than Unit S1 soils (Table 4). Moreover, water-soluble Mo, La, and Ce were higher in Unit S2 than Unit S1 and Mn in Unit S1 than Unit S2 (Table 4). In spite of available concentration, which shows no significant changes with depth, the water-soluble concentrations of Cr, Mn, Co, Ni, As, Y, In, Pr, and Zn tended to be lower while depth increased, especially for In, Mn, Co, and Zn which showed 11.6-, 7.98-, 3.20- and 2.59-times decreases from the surface (0–15 cm) to subsurface (15–30 cm) soils. The same result was reported by Martínez-Martínez et al. [65], who found that the concentrations of soluble Pb and Zn were higher in the surface layer than in the subsurface layer. When the mean of all data was evaluated, it showed that among all elements, Sb and Mn were found more in water-soluble fraction (2.87% and 0.82%, in comparison to total concentration), and Bi and Sn were not found in a water-soluble form (lower than detection limit) (Table 4). The water-soluble fraction of Pb, Cd, Mn, Cu, Sr, and Co decreased to less than 0.3% in comparison to the total concentration. These results indicate the lower mobility of these metals, assuming a lower risk of dispersion by runoff or leachate waters to the environment. 

The highlighted water-soluble Fe (2005–2024 µg kg^−1^, in Units S1 and S2), Pb (1419 µg kg^−1^ in Unit S2), and Zn and Al (1333 µg kg^−1^ in Unit S1) are still high, which indicates that these metals can be transported by water and pollute the surrounding areas and act as secondary contamination sources (Table 4). Martinez-Martinez et al. [65] have also reported the relatively high mobility of Zn in the soil environment. In addition, a negative correlation was found between clay content and water-soluble Mn, Co, Ni, Cu, Y, Cd, Ce, Pr, Ge, Rb, Zn, Fe, and Cr (Supplementary Material, Table S1). It is well known that the increase in clay content in the soil increases the number of cations sorbed by available sites [51] and reduces their mobility. However, this depends on the clay mineral(s) present in the soil clay fraction. Other soil properties such as CaCO_3_ content, other metals concentration, and soil organic carbon are also among important factors that may affect water-soluble forms of metal(loid)s and REEs in soils [63,65]. For example, Alvarez-Rogel et al. [66] found that water-soluble Cu was strongly correlated with water-soluble organic carbon, and the highest concentration of this metal in water extracts was found in a forest away from mine tailings soils, and a decrease in water-soluble Pb was observed when total Fe concentrations increased, while water-soluble Pb and total CaCO_3_ were uncorrelated [66].

### 2.3. Accumulation of Heavy Metals and Metalloids in Plant Tissues

**Figure 2:**
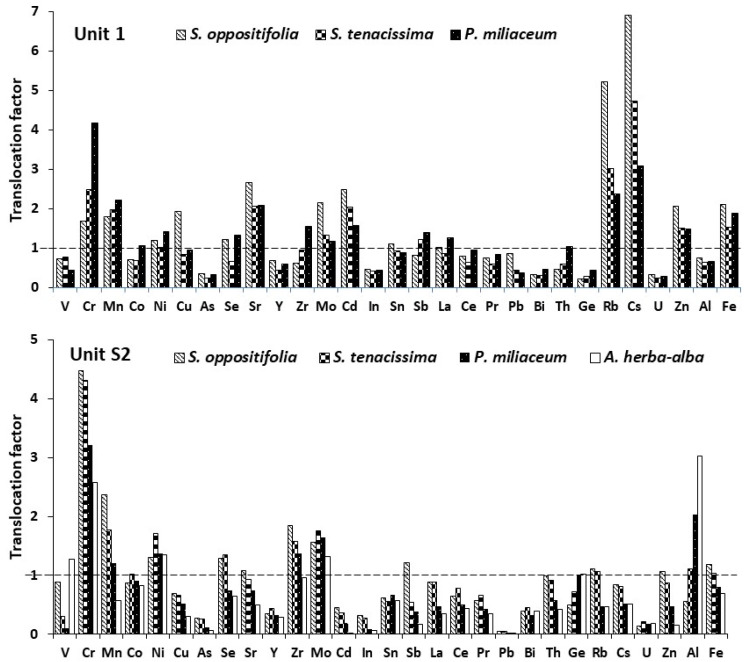
Translocation Factor (TF) for different metal(loid)s and REEs in Unit S1 and Unit S2.

The concentrations of different metal(loid)s and REEs in plant tissues are shown in Table 5, showing variable patterns of metal accumulation and distribution in their various parts and different soil units. Peñalver-Alcaraz et al. [15] and Martinez-Lopez et al. [18] reported that the growth of natural plants such as *Salsola* and *P. miliaceum* in harsh environments such as mine tailings that were polluted with metal(loid)s, indicating an adaptation and tolerance to contaminated conditions. The data showed the metal(loid) concentration in the plant tissues varied among species when compared in different units, indicating that the different capacities of plants for metal uptake could be affected by soil. The Zn concentration in plant shoots was found in variables ranging from deficient (*P. miliaceum* in Unit S2) to 547 mg kg^−1^ (*S. oppositifolia* in Unit 1), which is considered to be toxic according to Kabata-Pendias (100–400 mg kg^−1^) [67], and the maximum concentration was found in the root (687 mg kg^−1^) of *P. miliaceum* in Unit S1 (Table 5). The result of this research is in accordance with Ha et al. [68], who found a range of 13.3–380 mg kg^−1^ Zn accumulated in 21 native plant species grown in the soil near mining areas, with no toxicity symptoms. BCF_shoot_ > 1 and TF > 1 for Zn in *S. oppositifolia* revealed the effective absorption of Zn by roots and translocation to the aerial tissues, which could make this plant a candidate for the phytoextraction process only in Unit S1 (Table 6 and Figure 2). Therefore, this plant was successful in the mobilization of Zn into plant tissues and storage in the aerial plant biomass (TF > 1), but had difficulties in mobilizing Zn in the root zone of Unit S2 (BCF_root_ and BCF_root_ < 1).

Pb is not an essential element for plants, and its normal concentration is 5–10 mg kg^−1^; it becomes toxic to various plant species if it presents at 30–300 mg kg^−1^ in leaves [67]. The results showed that in *S. oppositifolia* and *P. miliaceum*, in Unit S1, Pb accumulated at a toxic level (31.4 and 31.1 mg kg^−1^, respectively) (Table 5). This should be considered the potential risk of incorporation of Pb into the food chain if local fauna feed on the leaves of *S. oppositifolia* and *P. miliaceum*. *P. miliaceum*, known as pioneer vegetation for the phytomanagement of metal(loid)-enriched tailings, could uptake 5.4 mg kg^−1^ Pb in leaves [26], which was lower than our result. 

The BCF_root_ and BCF_shoot_ index revealed that among all plants in the study area, *P. miliaceum* showed BCF_root_ > 1 for Pb in Unit S1, and these factors were smaller than the ones in other plant species, and TF were less than 1 in all cases; therefore, *P. miliaceum* can be considered interesting for phytostabilization of Pb in soil (Table 6 and Figure 2). Pb uptake studies in plants have demonstrated that roots have the ability to take up significant quantities of Pb, as in this study, *P. miliaceum* accumulated 1943 mg kg^−1^ in their roots, whilst it is simultaneously known as an immobile element in plant tissues, greatly restricting its translocation from root to shoot [33,69]. Similar to our study, Hasnaoui et al. [70] found that four plant species, *Reseda alba*, *Cistus libanotis*, *Stipa tenacissima*, and *Artemisia herba-alba* showed a strong capacity to tolerate and hyperaccumulate metal(loid)s, especially Pb, in their tissues.

There is no evidence of the essential role of Cr in plant metabolism (as there is in animals and humans), although plants accumulate it when it is available in the soil [36]. Considering the phytotoxic concentration of Cr (5–30 mg kg^−1^) in leaf tissue for different plant species reported by Kabata-Pendias [67], *P. miliaceum* in Unit S1 showed toxicity, which reflects the ability of this plant to adsorb and accumulate Cr. Total Cr concentration in plant shoot samples ranged from 1.03 mg kg^−1^ (*A. herba-alba* in Unit S2) to 6.61 mg kg^−1^ (*P. miliaceum* in Unit S1), with the maximum level in the root of *P. miliaceum* (9.18 mg kg^−1^) in Unit S2. In addition, *S. tenacissima* showed an approximately high concentration of Cr in their shoots (3.6 mg kg^−1^) in both Unit S1 and Unit S2 (Table 5). The BCF_shoot_ and TF revealed that in all plant species, BCF_shoot_ had Cr > 1 while the concentration of Cr in the shoots of *P. miliaceum*, *S. tenacissima*, and *S. oppositifolia* were 218, 79, and 50 times higher than those available in the soil of Unit S1, indicating a good capacity for bioaccumulation of Cr by these plants (Table 6 and Figure 2). Sinha et al. [71] reviewed that the reduction in Cr(VI) to Cr(III) by chemical or enzymatic processes, compartmentalization of Cr in the cytoplasm or in the vacuole, and phytochelatin-based sequestration are among the mechanisms that different plants acquired to cope with a high level of absorbed Cr. TF of Cr in all studied plants was higher than 1, and we concluded that these plants are proper candidates for phytoextraction of Cr in soil (Table 6 and Figure 2).

Cd is not an essential element in plant metabolic processes, and there is also no evidence of an essential role of Ni in plant metabolism, while Cu is an essential element for vegetation. When the available concentration of these metals increases in soil, they may be highly absorbed by plants, finally leading to toxicity in the plant. Kabata-Pendias [67] proposed the level of concentration of Cd, Ni, and Cu in leaves that caused toxicity to be 5–30, 10–100, and 20–100 mg kg^−1^, which in our study were lower in plants, since the highest concentrations of Cd and Cu were observed in *S. oppositifolia* in Unit S1 (3.48 and 15.5 mg kg^−1^, respectively) and Ni in *S. tenacissima* (6.57 mg kg^−1^) in Unit S2 that exceed the supraoptimal values of these elements proposed by Kabata-Pendias [67] (0.05–0.20, 5–30 and 0.10–0.50 mg kg^−1^, respectively). Moreover, except for Cd concentration in *P. miliaceum* in Unit S2 and Cu concentration in *S. tenacissima* and *P. miliaceum* in both soils, other plants showed more metal than the sufficient or normal concentration accepted in plant shoots [67] (Table 5). Midhat et al. [33] found that the majority of the collected plant species from three mining sites (Southern Centre Morocco) showed higher PTE concentrations than the normal or phytotoxic levels. They concluded that these plant species were tolerant to the studied PTEs surviving in soils with a high concentration of PTEs which are toxic to other plants, showing the ability to accumulate PTEs in their tissues without symptoms of toxicity [33]. 

The results revealed that for Ni, BCF_shoot_ was more than 1 in *S. oppositifolia* and *S. tenacissima*, and less than 1 in *A. herba-alba* while in *P. miliaceum* it was soil-dependent; that is, it was more than 1 in Unit S1 and less than 1 in Unit S2. Among different plants, only *S. oppositifolia* in Unit S2 showed BCF_shoot_ > 1 and TF > 1 for Cd and *A. herba-alba* and *P. miliaceum* in Unit S1 BCF_root_ > 1 and TF < 1 for Cd, which means they are suitable for phytoextraction and phytostabilization, respectively (Table 6 and Figure 2). Hasnaoui et al. [70], in the screening of native plants growing on a Pb/Zn mining area in eastern Morocco, found that BCF_root_, BCF_shoot_, and TF for Cd in *S. tenacissima* and *A. herba-alba* were 2.72–0.80, 0.32–1.10, and 0.11–1.11, respectively. TF for Ni was more than 1 in all plants and soils except that of *P. miliaceum* in Unit S2. Briefly, while *S. oppositifolia* and *S. tenacissima* are good candidates for Ni phytoextraction, *A. herba-alba* is not a proper candidate in this regard, and *P. miliaceum* could phytoextract Ni only in Unit S1 (Table 6 and Figure 2). Hasnaoui et al. [65] reported that in their study BCF_root_, BCF_shoot_, and TF for Ni in *S. tenacissima* and *A. herba-alba* were 0.40–0.54, 0.23–0.62, 0.59–1.24, respectively. *S. oppositifolia*, *S. tenacissima*, and *P. miliaceum* in Unit S1 and *A. herba-alba* in Unit S2 showed BCF_root_ > 1 and TF < 1 for Cu, and could be a candidate for phytostabilization. Moreover, *S. oppositifolia* in Unit S1 was the only plant that had BCF_shoot_ > 1 and TF > 1, and was suitable for the phytoextraction of Cu from soil (Table 6 and Figure 2). Hasnaoui et al. [70] reported that in their study BCF_root_, BCF_shoot_, and TF for Cu in *S. tenacissima and A. herba-alba* were 1.55–3.36, 0.19–1.29, and 0.12–0.35, respectively.

The results obtained from plant analysis showed that the concentration of V, As, Co, Mn, Mo, Se, and Sb in the shoots of plants are in the sufficient or normal range proposed by Kabata-Pendias [67] (Table 5). In the average of two Unit soils, the minimum and maximum of V and As found in *P. miliaceum* and *A. herba-alba* (614–1380 and 947–7144 µg kg^−1^, respectively), Mn, Co, and Se in *S. oppositifolia* and *A. herba-alba* (18,993–136,394, 102–351 and 47.3–67.6 µg kg^−1^, respectively) while *A. herba-alba* showed the maximum (663 µg kg^−1^) and *S. tenacissima* minimum (306 µg kg^−1^) concentration of Mo (Table 5). Different species from the *Salsola* genera are the major species in semiarid environments due to their fast growth, large biomass, drought tolerance, and universal adaptability, including in extremely harsh environments, and play an important role in phytoremediation processes of different metal(loid)s such as Co, Fe, Mn, Sr, As, V, Mo, and Cd [24]. According to data from TF, BCF_root,_ and BCF_shoot_, it can be concluded that *A. herba-alba* has the ability for the phytoextraction of V (TF > 1 and BCF_shoot_ > 1), while other plants could stabilize this element (TF < 1 and BCF_root_ > 1). All plants accumulate high amounts of As in their roots (BCF_root_ for *P. miliaceum*, *S. tenacissima*, and *S. oppositifolia* were 790, 80, and 69, respectively, in Unit S1) except for *S. tenacissima* in Unit S1; therefore, these native plant species with both the capacity to accumulate high amounts of As in their roots and have low values of the translocation from root to shoot (TF < 1), could be used to minimize the migration of As in soil (Figure 2). Based on BCF_root_, TF, and BCF_shoot_ values, none of the plant species have the potential to be used in Mn and Co phytoextraction or phytostabilization except for *S. oppositifolia*, which has the potential to extract Mn (BCF_shoot_ and TF were 3.44 and 1.80, respectively) and stabilize Co (BCF_root_ and TF were 1.47 and 0.70, respectively) in Unit S1 (Figure 2). The high BCF_shoot_ values for Mo in *S. oppositifolia* (29.6), *S. tenacissima* (11.5), and *P. miliaceum* (21.2), with TF > 1 (5.09, 1.52, and 1.44, respectively), were measured, which then suggested these plants were suitable for Mo phytoextraction from the soil. However, the BCF_root_ >1 and TF < 1 of Mo (19.08) in *A. herba-alba* showed their high ability to tolerate and accumulate Mo in their roots, suggesting they are suitable for Mo phytostabilization (Table 6 and Figure 2). 

In our study, total Al concentration in plant samples ranged from 578 mg kg^−1^ (*S. tenacissima* in Unit S1) to 41.5 mg kg^−1^ (*S. oppositifolia* in Unit S1), with the maximum level in the roots of *S. tenacissima* (750 mg kg^−1^) in Unit S1 and the roots of *P. miliaceum* (660 mg kg^−1^) in Unit S2 (Table 5). Considering the mean concentration of Al (10–1000 mg kg^−1^) in leaf tissue for different plant species reported by Kabata-Pendias [67], in all plants, the Al concentration of shoots was in the given range, which means there is not any risk of toxicity entering the food chain. However, Aluminum toxicity is an important growth-limiting factor for plants in acid soils, especially below pH 5.0 [36]; the alkaline pH of the study area leads to a decrease in Al availability, despite the high total concentration in the soil (Table 2 and Table 4). BCF_root_ and BCF_shoot_ in all plants were higher than 1 (maximum for *S. tenacissima* were 116 and 55.3 in Unit S1, respectively (Table 6 and Figure 2), while TF was variable for plants according to soil unit (e.g., in the case of *S. tenacissima* TF < 1 in Unit S1 and TF > 1 in Unit S2), and only in the case of *S. oppositifolia*, it was less than 1 in both soils, so this plant has the ability to stabilize Al in both Units (Table 6 and Figure 2).

The result of REEs accumulation in plants revealed that Y, La, Ge, and Pr concentration in shoot showed maximum levels in *S. oppositifolia* and *P. miliaceum* in Unit S1 (512–517, 497–734, 133–261, and 110–198 µg kg^−1^, respectively), Ce in *S. oppositifolia*, *S. tenacissima*, and *P. miliaceum* in Unit S1 (519, 542 and 1307 µg kg^−1^, respectively) and Th in *P. miliaceum* (421 µg kg^−1^) in Unit S1 (Table 5). Wiche and Heilmeier [72] found the mean concentrations of La as representative for the REEs ranged from 24 to 146 ng g^−1^ in grasses (*Hordeum vulgare*, *Zea mays*, *Avena sativa*, *P. miliaceum*, and *Phalaris arundinacea*) and 20–250 ng g^−1^ in herbs (*Lupinus albus*, *Lupinus angustifolius*, *Fagopyrum esculentum*, and *Brassica napus*) when grown in soil from a road construction site or mining affected area containing 25–26 µg g^−1^ total La. With the exception of hyperaccumulating plants, the content of REEs in plants is generally very low [47]. In general, the concentration of REEs in plants is influenced by several factors, namely soil properties, e.g., pH and total C, cation exchange capacity, redox potential, availability and nature of other elements, e.g., N, P, Ca, Mg, and Al, and plant species, especially in regard to root characteristics [47]. For instance, treatment of soil with organic material (OM) may inhibit REE uptake by *Phytolacca Americana* through competition between cations (e.g., Ca and Mg) and REEs, and adding biochar to soil may avoid the extensive precipitation of P and REEs on the root surface and root apoplast, thereby promoting REE uptake by plants [73]. Similarly, Saatz et al. [74] found significant correlations between the concentration of Gd and Y in the nutrient solution and the root tissue concentration of Ca, Mg, and P. The result of TF showed that *A. herba-alba* had TF > 1 for La, Ce, Pr, Y, and TF < 1 for Th; *S. oppositifolia* in Unit S1 had TF > 1 for La, and other plants had TF < 1 for all REEs (Figure 2). Under normal circumstances, the TF of REEs is less than 1, except for hyperaccumulating ones [47,75]. By considering if BCF_root_ > 1 and TF < 1, the plant could be a good candidate for phytostabilization, and BCF_shoot_ > 1 and TF > 1 could select the plant for phytoextraction; it was found that for La, *S. oppositifolia*, *S. tenacissima*, and *P. miliaceum* had a BCF_root_ of 1.45, 3.28, and 5.86, respectively, and could stabilize La in the root. For Ce, *S. tenacissima* and *P. miliaceum* had a BCF_root_ of 2.14 and 5.26, for Th *S. oppositifolia*, *S. tenacissima* and *P. miliaceum* had a BCF_root_ of 3.6, 6.74, and 10.1, and for Pr *S. oppositifolia*, *S. tenacissima* and *P. miliaceum* had a BCF_root_ of 1.25, 3.85, and 5.18, and therefore, these native plant species with both the capacity to accumulate these REEs in their roots and low values of the translocation from root to shoot could be used for phytostabilization. None of the plant species has the potential to be used in phytoextraction of REEs and/or phytostabilization of Y (Table 6 and Figure 2). The selective absorption of root cell walls (in the form of trivalent cations) and the co-precipitation of rare earth ions-salts (mostly in the form of insoluble oxalates or phosphates) are the main mechanisms through which plant roots fix REEs [76]. When REEs come in contact with plant roots, rare earth cations combine with free carboxyl groups such as cellulose and pectin on the cell wall, and the positive and negative charges attract each other, resulting in selective absorption by the cell wall [47,77].

## 3. Materials and Methods

### 3.1. Site Characterization

**Figure 3:**
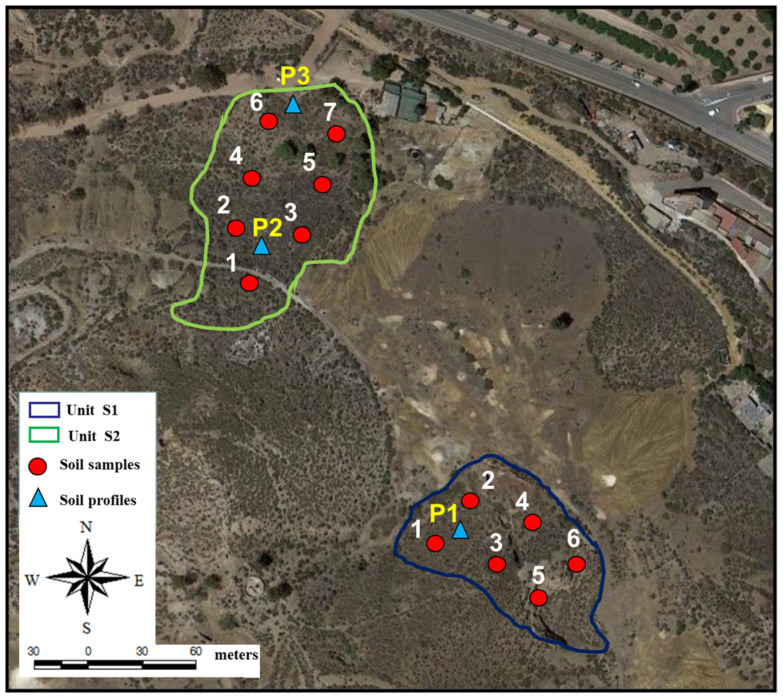
Study area and sampling points.

The plant and soil samples used in this study were collected from two natural soil sites (Units S1 and S2) located in a mining area at the extreme northeast of the Cabezo de San Cristobal, in the municipality of Mazarron (Murcia Region, SE Spain), 20 m from the urban area. The mining activity for Pb, Ag, and Zn extraction had been carried out for hundreds of years in this area and was abandoned in 1996, while many tailing ponds remain without any restoration (Figure 3) [28].

Unit S1 is an area with natural soil without the presence of mining waste and is located south of the main waste pond of greater volume and extension, made up of gravimetric waste (>50 cm) covered by sludge waste (0–50 cm deep). Unit S2 is a large area with natural soil without the presence of mining waste and is located near a tailing pond made up of gravimetric waste (>30 cm) covered by industrial sludge waste (0–30 cm deep) (Figure 3). The climate of the area is semiarid Mediterranean with an average monthly temperature of 18 °C, and a mean annual precipitation of 350 mm, with rainfall events occurring mostly in autumn and spring; the most important winds in summer are from the E, and the prevailing winds are from the N and NE in winter [28].

For this study, a vegetation census was carried out in each environmental Unit, identifying the different species found and the vegetation cover. Unit S1 presented a plant coverage of 80%, dominated by *Salsola oppositifolia*, *Stipa tenacissima*, and *Piptatherum miliaceum*, indicating a high degree of degradation of the potential plant community, with the presence of *Hyparrhenia sinaica*, *Artemisia herba-alba*, *Capparis spinosa*, *Angustifolia Route*, *Asparagus horridus*, *Whitania frutescens*, and *Dianthus broteri*. In addition, Unit S2 presents a plant coverage of 80% as well, dominated by *Salsola oppositifolia*, *Stipa tenacissima*, *Piptatherum miliaceum*, and *Artemisia herba-alba*, indicating a high degree of degradation of the potential plant community, with the presence of *Whitania frutescens*, *Ballota hirsute*, *Angustifolia Route*, *Asparagus horridus*, *Avena sativa*, *Hyparrhenia sinaica*, *Dianthus broteri*, *Thymelaea hirsute*, *Sedum sediforme*, *Launaea arborescens*, *Pinus halepensis*, and *Salsola genistoides.* The high vegetation cover contributes to soil conservation and prevents soil loss due to erosion.

### 3.2. Soil Sampling and Analysis

In order to evaluate the actual impact of the mine tailings on natural soils around them, different samples were collected from Unit S1 and Unit S2 (6 and 7 samples, respectively) at surface (0–15 cm depth) and subsurface (15–30 cm depth). Moreover, to characterize the study area edaphologically and analyze the vertical variation in the natural areas and the possible impact by various geomorphologic, edaphic, and lithologic characteristics, external geological agents, and anthropic activities, three representative soil profiles have been taken, one in Unit S1, and two in Unit S2. The soil profiles examined in Units S1 and S2 had three horizons: A1 (0–10 cm), A2 (10–20 cm), C/R (20–50 cm) for S1, and A (0–10 cm), AC (10–20 cm), C/R (30–40 cm) and A (0–20 cm), C1 (20–35 cm), C2 (35–50 cm) for S2.

All soil samples were oven dried at 40 °C for 48 h, passed through a 2 mm stainless steel sieve to remove stones and roots, and crushed and stored in plastic bags at room temperature prior to laboratory analysis. Soil pH values were determined with a pH meter at a soil: water ratio of 1:2.5 (*w*/*v*) [78]. Electrical conductivity (EC) was analyzed using EC meter after extraction with water (1: 5, *w*/*v*) [78]. The content of clay (<0.002 mm), silt (0.02–0.002 mm), and sand (2–0.02 mm) in the fine soil samples (grains < 2 mm in diameter) were determined by the Bouyoucos method to identify soil texture [79]. In addition to these parameters, in the samples from the soil profiles, total organic carbon (TOC) and total nitrogen (TN) by CNHS-O elemental analyzer (EA-1108, Carlo Erba, Cornared, Italy), cation exchange capacity (CEC) through the use of barium chloride as a saturating agent of the soil change complex [80], and equivalent calcium carbonate by means of Bernard’s calcimeter, were determined.

Soil samples were digested using a mixture of strong acids (nitric-perchloric acid) while heating for 40 min to determine total content of metals, metalloids, and rare earth elements (REEs) [81]. Twenty-nine selected elements (V, Cr, Mn, Co, Ni, Cu, As, Se, Sr, Y, Zr, Mo, Cd, In, Sn, Sb, La, Ce, Pr, Bi, Th, Ge, Rb, Cs, Pb, Zn, Al, Fe, and U) in the filtrated extractants were measured by mass spectrometry with an inductively coupled plasma source ICP-MS (Agilent 7500 CE). Bioavailable and water-soluble contents of metal(loid)s and REEs were measured by extraction of soil samples by organic acid DTPA (diethylene triamine-pentaacetic acid), 0.05 M adjusted to pH 7.5 [82], and the application of deionized water to the sample in a 1:5 ratio, in which soluble metals are extracted by continuous stirring for 6 h. The selected elements in filtered extractants were measured as discussed above. The detection limits of the element measured were (μg L^−1^): V (0.15), Cr (0.61), Mn (0.03), Co (0.14), Ni (1.18), Cu (1.08), As (0.91), Se (0.41), Sr (1.24), Y (0.17), Zr (0.03), Mo (0.11), Cd (0.14), In (0.01), Sn (0.11), Sb (2.08), La (0.09), Ce (0.19), Pr (0.04), Bi (0.05), Th (0.03), Ge (0.11), Rb (0.37), Cs (0.05), Pb (7.71), Zn (0.72), Al (1.31), Fe (1.35), and U (0.18). Certified reference material (BAM-U110) from the Federal Institute for Materials Research and Testing and reagent blanks were used as quality control samples during the analyses. The recovery of As, Cd, Co, Cr, Cu, Mn, Ni, Pb, and Zn in the analysis was within <7.0% compared to this reference sample. No reference material was used for the rest of the elements analyzed.

### 3.3. Plant Sampling and Analysis

In order to estimate the current risk of bioaccumulation and transfer of toxic elements in the trophic chain, as well as to evaluate the potential of native vegetation to stabilize contaminated soils, the most abundant plant species that grow in the study area have been selected. The species selected for their abundance were *Salsola oppositifolia*, *Stipa tenacissima*, *Piptatherum miliaceum* from both Units S1 and S2, and *Artemisia herba-alba* from Unit S2. Three random specimens of each species were uprooted from Unit S1 and Unit S2 [28,83,84,85]. In order to preserve the root system during sampling, a deeper and wider hole than the space occupied by the root system of each plant was excavated. Subsequently, the whole plant (including the root system) was carefully extracted from the soil. Then, roots and the aerial part (shoots) were separated and stored in polyethylene bags for their transport. 

The different plant parts were transported to the laboratory and carefully washed with deionized water. They were then dried in an oven at 55 °C for 72 h. The dried material was ground using a mill (A11 Basic, IKA, Staufen, Germany). For each sample, 0.7 g were weighed, which were incinerated in a muffle furnace at 450 °C for 12 h. Subsequently, the metals and metalloids were dissolved in 0.6 N HNO_3_, and the extracts were stored at 4 °C until analysis. The elements, as in soils, were measured by mass spectrometry with an inductively coupled plasma source ICP-MS (Agilent 7500 CE, Santa Clara, CA, USA).

### 3.4. Data Treatment and Statistical Analyses

#### 3.4.1. Contamination, Bioconcentration, Translocation, and Accumulation Factors 

Several indices have been used to study the degree of pollution in contaminated soils, such as concentration factors (CF), enrichment factors (EF), and pollution load index (PLI) [27,46]. In this study, the contamination factor (CF), the ratio of PTEs and REEs concentration in the soil in study area to that of the reference value, was used for the assessment of soil contamination.

(1) $$\mathrm{CF}=\frac{\left(Metal/RE\right)\;soil}{\left(Metal/RE\right)\;\mathrm{reference}\;\mathrm{value}}$$

According to Hakanson [86], if CF < 1, soil quality is classified as low contamination, 1 ≤ CF < 3 as moderate contamination, 3 ≤ CF < 6 as considerable contamination, and 6 ≤ CF as very high contamination. In our study, we applied the concentration of elements in the reference levels proposed for the Murcia Region, SE Spain [53,87]. In case there were no data on any particular element for the Murcia Region, the background levels and generic reference levels published by the Geological and Mining Institute of Spain for the autonomous community of Aragon, NE Spain [54] and Ballesta et al.’s [56] reference levels proposed for Castilla La Mancha (Central Spain) were utilized (Table 7).

In order to evaluate the natural plants in study areas for their ability to extract or stabilize metal(loid)s and REEs in soil, the bioaccumulation factor (BCF) of roots and shoots (Equations (2) and (3)) and transfer factor (TF) (Equation (4)) were calculated. It was suggested that the plants exhibiting greater than 1 BCF_root_ and less than 1 TF (translocation factor) could be used as potential candidates for the phytostabilization, while plants with BCF_shoot_ and TF both greater than one have the potential to be used for phytoextraction [16,17].

BCF_root_ = (Metal(loid)/REE) root/bioavailable (Metal(loid)/REE) soil (2)

BCF_shoot_ = (Metal(loid)/REE) shoot/bioavailable (Metal(loid)/REE) soil (3)

TF = (Metal(loid)/REE) shoot/(Metal(loid)/REE) root(4)

Moreover, to carry out the analysis of risks and potential for phytostabilization of the sampled species, the concentrations of metal(loid)s and REEs have been compared with the toxicity thresholds, when they exist, for vegetation and herbivores proposed by Kabata-Pendias [67].

#### 3.4.2. Statistical Analysis

All data were tested for goodness of fit to a normal distribution using a Kolmogorov–Smirnov method prior to statistical analysis. Data were log transformed to achieve homogeneity of variance where the distribution was not normal [27]. The evaluation of significant differences between different data was carried out using ANOVA test followed by a Duncan test (*p* ≤ 0.05), with the SAS 9.4 software. The correlation among soil properties and metal(loid) and REE concentrations was determined using Spearman correlation coefficients using IBM SPSS Statistics 20 software.

## 4. Conclusions

This study emphasizes the importance of paying attention to abandoned mines and managing mine tailings because they can cause pollution to enter the surrounding soils. Results indicated that the degree of pollution of metal(loid)s and REEs in natural soils near mine areas is heterogeneous, and therefore, before starting remediation programs, a study of the spatial distribution of toxic elements in soil should be performed in order to identify the most polluted areas, in which more attention should be paid for their remediation. As shown, plants grown in soils with a higher concentration of toxic elements contained more metal(loid)s in their tissues, showing a higher phytoextraction ability of metal(loid)s, such as Cr, Ni, Zn, and Cd, which could pass into the food chain via herbivores, and thus provide more potential risk to humans. The results suggested that among the four species studied (*S. oppositifolia*, *S. tenacissima*, *P. miliaceum*, and *A. herba-alba*), the suitability of the plant for stabilization or extraction of metal(loid)s completely depends on the type of metal(loid)s and soil type. Although *A. herba-alba* was the least efficient plant in the phytoremediation process, *P. miliaceum* was a good candidate for phytostabilization of Pb, Cd, Cu, V, and As, and *S. oppositifolia* was a good candidate for phytoextraction of Zn, Cd, Mn, and Mo. All plant species except *A. herba-alba* could be potential candidates for the phytostabilization of REEs, while none of the plant species has the potential to be used in the phytoextraction of REEs. Therefore, the use of native plants is a recommended method for reclaiming polluted mine soils; the selection of the plant species to be used is a key factor for the success of the phytoremediation strategy.

## Figures and Tables

**Table 1:** Geochemical properties of soil in studied area.

	Horizon	Depth	pH	EC *	OC *	TN *	CEC *	Carbonates	Sand	Silt	Clay	Soil Texture
		cm		dS m^−1^	%	%	cmol^+^ kg^−1^	%				
**Profile 1**	**A1**	0–10	7.67	0.347	3.1	0.24	9.36	2.38	61	18	21	Sandy Clay Loam
	**A2**	10–20	8.13	0.180	1.8	0.15	8.73	8.05	62	12	26	Sandy Clay Loam
	**C/R**	20–50	8.26	0.246	0.9	0.09	8.3	10.64	66	10	24	Sandy Clay Loam
**Profile 2**	**A**	0–10	8.04	0.591	2.4	0.2	8.9	0.84	62	10	28	Sandy Clay Loam
	**AC**	10–20	8.18	0.480	1.7	0.15	9.87	15.02	66	12	22	Sandy Clay Loam
	**C/R**	20–40	8.18	0.503	0.8	0.1	10.01	19.71	78	8	14	Sandy Loam
**Profile 3**	**A**	0–20	7.92	0.298	3.1	0.19	9.48	6.36	68	10	22	Sandy Clay Loam
	**C1**	20–35	8.26	0.235	0.9	0.1	9.63	32.64	59	9	32	Sandy Clay Loam
	**C2**	35–50	7.97	0.773	0.8	0.06	9.28	43.05	49	9	42	Sandy Clay
**Unit S1**	**Surface**	0–15	7.86 b	0.168 a	-	-	-	-	64	12	24	Sandy Clay Loam
	**Sub-surface**	15–30	7.97 b	0.138 a	-	-	-	-	62	13	25	Sandy Clay Loam
**Unit S2**	**Surface**	0–15	8.13 a	0.190 a	-	-	-	-	62	15	23	Sandy Clay Loam
	**Sub-surface**	15–30	8.27 a	0.193 a	-	-	-	-	57	14	29	Sandy Clay Loam

* EC: electrical conductivity; OC: organic carbon; TN: total nitrogen; CEC: cation exchange capacity. Different letters indicate significant differences (*p* < 0.05) between means after an ANOVA test.

**Table 2:** Total concentrations of metals and REEs (mg kg^−1^) in surface (0–15 cm) and subsurface (15–30 cm) soils from Units S1 and S2.

	Unit S1			Unit S2			F Value		
	Surface Soil	Subsurface Soil	Mean	Surface soil	Subsurface Soil	Mean	Unit	Depth	Unit × Depth
**V**	81.1 ± 10.6 ab	86.8 ± 5.2 a	83.9 A	76.1 ± 9.7 ab	68.5 ± 13.5 b	72.3 B	5.87 *	0.04 ns	1.93 ns
**Cr**	102 ± 47.2 a	119 ± 52 a	110 A	92.2 ± 18.2 a	80.9 ± 15.6 a	86.5 A	3.00 ns	0.04 ns	1.01 ns
**Mn**	369 ± 121 b	287 ± 192 b	328 B	795 ± 254 a	593 ± 308 ab	694.5 A	8.48 *	1.26 ns	0.23 ns
**Co**	4.70 ± 0.42 c	4.50 ± 1.37 c	4.60 B	13.4 ± 3.6 a	10.0 ± 1.4 b	11.7 A	32.34 **	2.09 ns	1.66 ns
**Ni**	28.5 ± 3.76 b	40.6 ± 19.8 ab	34.5 A	45.3 ± 14.0 a	30.9 ± 3.6 b	38.1 A	0.46 ns	0.04 ns	6.29 *
**Cu**	46.5 ± 14.2 b	33.1 ± 13.7 b	39.8 B	82.2 ± 28.7 a	59.9 ± 21.3 ab	71.1 A	8.37 *	2.75 ns	0.17 ns
**As**	130 ± 12 a	131 ± 54 a	131 A	153 ± 35 a	144 ± 41 a	149 A	0.99 ns	0.05 ns	0.07 ns
**Se**	1.87 ± 1.12 ab	2.44 ± 1.04 a	2.16 A	1.42 ± 0.39 b	1.22 ± 0.23 b	1.32 B	7.95 *	0.42 ns	1.72 ns
**Sr**	130 ± 64 b	140 ± 79 b	135 B	484 ± 106 a	434 ± 93 a	459 A	50.13 **	0.19 ns	0.43 ns
**Y**	22.7 ± 2.9 ab	24.92 ± 9.99 a	23.8 A	16.8 ± 3.8 bc	13.1 ± 1.5 c	14.9 B	17.99 **	0.13 ns	1.91 ns
**Zr**	4.72 ± 0.88 b	5.20 ± 2.14 b	4.96 B	16.1 ± 2.1 a	15.4 ± 3.2 a	15.8 A	70.04 **	0.03 ns	0.19 ns
**Mo**	3.39 ± 0.94 a	3.48 ± 0.92 a	3.44 A	2.82 ± 1.11 a	1.63 ± 0.38 b	2.22 B	9.80 *	1.97 ns	2.78 ns
**Cd**	15.4 ± 6.2 a	12.8 ± 6.7 ab	14.1 A	7.21 ± 2.08 bc	6.13 ± 2.48 c	6.67 B	15.31 *	0.95 ns	0.17 ns
**In**	1.62 ± 0.81 a	1.18 ± 0.86 ab	1.40 A	0.93 ± 0.34 ab	0.81 ± 0.39 b	0.87 A	4.32 *	1.17 ns	0.42 ns
**Sn**	5.93 ± 1.48 ab	7.80 ± 2.63 a	6.87 A	6.55 ± 4.96 a	2.84 ± 0.86 b	4.70 A	2.07 ns	0.37 ns	3.42 ns
**Sb**	2.99 ± 1.05 a	3.12 ± 0.42 a	3.06 A	5.00 ± 1.81 a	3.39 ± 0.91 a	4.20 A	1.92 ns	0.80 ns	1.11 ns
**La**	41.9 ± 5.1 b	66.4 ± 6.6 a	54.1 A	28.9 ± 4.1 c	23.6 ± 4.0 d	26.3 B	153.59 **	18.22 **	43.93 **
**Ce**	92.6 ± 9.3 b	144 ± 14 a	118 A	65.9 ± 9.1 c	56.3 ± 9.7 c	61.2 B	136.11 **	18.54 **	39.06 **
**Pr**	12.4 ± 1.5 b	18.2 ± 1.64 a	15.3 A	8.43 ± 1.21 c	7.34 ± 1.27 c	7.89 B	137.35 **	13.46 *	29.03 **
**Bi**	0.37 ± 0.17 ab	0.20 ± 0.01 b	0.29 A	0.56 ± 0.27 a	0.38 ± 0.21 ab	0.47 A	3.19 ns	2.44 ns	0.01 ns
**Th**	25.5 ± 3.5 b	47.9 ± 4.8 a	36.7 A	18.4 ± 3.8 c	16.2 ± 2.7 c	17.3 B	109.27 **	29.49**	43.80 **
**Ge**	4.57 ± 0.09 b	5.31 ± 0.19 a	4.94 A	3.10 ± 0.57 c	2.56 ± 0.52 d	2.84 B	112.59 **	0.28 ns	10.41*
**Rb**	61.2 ± 13.61 a	70.8 ± 4.3 a	66.0 A	71.5 ± 21.8 a	76.0 ± 29.5 a	73.8 A	0.49 ns	0.40 ns	0.05 ns
**Cs**	6.63 ± 1.51 b	7.14 ± 0.54 b	6.89 B	38.7 ± 20.1 a	46.5 ± 19.6 a	42.6 A	18.05 **	0.25 ns	0.19 ns
**U**	8.32 ± 1.62 a	7.52 ± 1.78 a	7.92 A	4.45 ± 0.65 b	4.08 ± 0.74 b	4.26 B	53.67 **	1.35 ns	0.19 ns
**Fe**	63,354 ± 6764 a	53,283 ± 4005 a	58,318 A	40,828 ± 6003 b	35,988 ± 8450 b	38,408 B	34.74 **	4.85 *	0.60 ns
**Pb**	2985 ± 1655 a	1704 ± 1612 a	2345 A	2613 ± 920 a	2443 ± 1134 a	2528 A	0.10 ns	1.54 ns	0.91 ns
**Zn**	2533 ± 440 a	2338 ± 289 a	2436 A	1283 ± 300 b	1091 ± 366 b	1187 B	51.54 **	1.23 ns	0.00 ns
**Al**	44,179 ± 1791 a	52,831 ± 3089 a	48,505 A	29,763 ± 5490 b	26,491 ± 6692 b	28,127 B	58.60 **	1.02 ns	5.02 *

Different letters indicate significant differences (*p* < 0.05) between means after an ANOVA test. * *p* < 0.05; ** *p* < 0.01; ns: non-significant (*p* > 0.05).

**Table 3:** Available concentrations of metals and REEs (mg kg^−1^) in surface (0–15 cm) and subsurface (15–30 cm) soils from Units S1 and S2.

	Unit S1			Unit S2			F Value		
	Surface Soil	Subsurface Soil	Mean	Surface Soil	Subsurface Soil	Mean	Unit	Depth	Unit × Depth
**V**	17.9 ± 6.5 b	12.3 ± 10.4 b	15.1 B	81.5 ± 25.3 a	72.5 ± 16.9 a	77.0 A	43.74 **	0.61 ns	0.03 ns
**Cr**	62.6 ± 69.0 ab	10.5 ± 2.3 b	36.6 A	54.2 ± 56.5 ab	136 ± 115 a	95.3 A	2.14 ns	0.14 ns	2.80 ns
**Mn**	39.2 ± 7.1 a	39.6 ± 12.1 a	39.4 A	44.4 ± 34.9 a	26.0 ± 15.7 a	35.2 A	0.13 ns	0.59 ns	0.65 ns
**Co**	221 ± 122 a	228 ± 129 a	225 A	417 ± 318 a	324 ± 197 a	371 A	1.58 ns	0.14 ns	0.19 ns
**Ni**	277 ± 60 a	364 ± 270 a	321 A	434 ± 117 a	373 ± 163 a	403 A	1.15 ns	0.03 ns	0.92 ns
Cu *	4.14 ± 1.98 ab	2.17 ± 1.59 b	3.16 A	5.53 ± 2.31 a	4.15 ± 1.51 ab	4.84 A	3.23 ns	3.20 ns	0.10 ns
**As**	20.4 ± 2.1 b	10.1 ± 4.8 b	15.3 B	80.4 ± 24.1 a	60.5 ± 24.6 a	70.5 A	28.40 **	2.12 ns	0.21 ns
**Se**	30.8 ± 13.8 a	20.8 ± 12.2 ab	25.8 A	14.0 ± 10.3 b	12.4 ± 11.4 b	13.2 B	5.06 ns	1.08 ns	0.58 ns
Sr *	7.83 ± 1.52 b	9.26 ± 1.75 b	8.54 B	11.2 ± 2.3 ab	15.2 ± 5.0 a	13.2 A	7.45 *	2.49 ns	0.55 ns
**Y**	1.28 ± 0.35 a	0.90 ± 0.53 a	1.09 A	0.33 ± 0.14 b	0.24 ± 0.20 b	0.28 B	36.19 *	3.05 ns	1.17 ns
**Zr**	6.93 ± 2.95 a	6.67 ± 2.48 a	6.80 A	10.2 ± 4.8 a	7.77 ± 6.32 a	8.99 A	0.78 ns	0.30 ns	0.20 ns
**Mo**	13.8 ± 1.7 a	10.5 ± 5.7 a	12.1 A	17.3 ± 7.3 a	17.3 ± 4.9 a	17.3 A	3.24 ns	0.33 ns	0.32 ns
Cd *	3.09 ± 1.61 a	2.17 ± 1.63 a	2.63 A	0.83 ± 0.42 a	0.61 ± 0.45 a	0.72 B	19.15 **	1.70 ns	0.65 ns
**In**	3.80 ± 1.69 a	1.84 ± 1.62 a	2.82 A	2.17 ± 2.65 a	1.61 ± 1.43 a	1.89 A	0.89 ns	1.61 ns	0.49 ns
**Sn**	nd	nd	nd	nd	nd	nd	-	-	-
**Sb**	64.5 ± 22.3 b	44.6 ± 39.8 b	54.6 B	143 ± 59 ab	180 ± 95 a	162 A	9.72 *	0.06 ns	0.69 ns
**La**	269 ± 120 a	105 ± 57 b	187 A	223 ± 95 ab	142 ± 79 ab	182 A	0.01 ns	7.85 ns	0.91 ns
Ce *	0.62 ± 0.05 a	0.32 ± 0.19 a	0.47 A	0.82 ± 0.46 a	0.60 ± 0.45 a	0.71 A	1.54 ns	1.74 ns	0.03 ns
**Pr**	88.5 ± 38.0 a	36.8 ± 21.1 a	62.6 A	70.4 ± 30.2 a	52.1 ± 39.1 a	61.3 A	0.01 ns	4.46 ns	1.02 ns
**Bi**	8.40 ± 3.5 a	5.17 ± 5.76 a	6.79 A	11.1 ± 10.7 a	6.38 ± 5.35 a	8.75 A	0.27 ns	1.11 ns	0.04 ns
**Th**	13.6 ± 1 a	10.6 ± 4.02 a	12.1 A	16.2 ± 7.9 a	12.0 ± 6.96 a	14.1 A	0.36 ns	1.23 ns	0.03 ns
**Ge**	25.9 ± 7.7 a	15.0 ± 8.42 a	20.5 A	51.0 ± 37.8 a	21.6 ± 17.5 a	36.3 A	1.57 ns	2.55 ns	0.54 ns
**Rb**	19.7 ± 6.3 b	21.8 ± 1.84 b	20.8 B	58.6 ± 24.3 a	34.3 ± 18.00 a	46.4 A	7.92 *	1.48 ns	2.11 ns
**Cs**	ndb	ndb	nd B	0.63 ± 0.90 ab	1.29 ± 0.55 a	0.96 A	9.19 *	1.09 ns	1.09 ns
**U**	9.82 ± 7.07 a	10.1 ± 14.5 a	9.97 A	6.38 ± 2.54 a	7.56 ± 4.31 a	6.97 A	0.90 ns	0.06 ns	0.02 ns
Fe *	9.78 ± 2.55 a	6.18 ± 1.93 ab	7.98 A	8.20 ± 4.05 a	4.93 ± 1.10 b	6.56 A	1.07 ns	6.31 ns	0.01 ns
Pb *	581 ± 351 a	329 ± 33 a	455 A	346 ± 208 a	337 ± 228 a	342 A	0.83 ns	1.10 ns	0.96 ns
Zn *	56.9 ± 13.0 a	36.3 ± 26.7 a	46.6 A	57.5 ± 30.6 a	50.1 ± 33.7 a	53.8 A	0.24 ns	0.92 ns	0.20 ns
Al *	7.62 ± 7.41 a	2.59 ± 3.30 ab	5.11 A	4.35 ± 2.33 ab	1.67 ± 0.93 b	3.01 A	1.74 ns	5.87 ns	0.54 ns

Elements identified with * are represented in mg kg^−1^ and other elements with µg kg^−1^. Nd non-detected. Different letters indicate significant differences (*p* < 0.05) between means after an ANOVA test. * *p* < 0.05; ** *p* < 0.01; ns: non-significant (*p* > 0.05).

**Table 4:** Water-soluble concentrations of metals and REEs (mg kg^−1^) in surface (0–15 cm) and subsurface (15–30 cm) soils from Units S1 and S2.

	Unit S1			Unit S2			F Value		
	Surface Soil	Subsurface Soil	Mean	Surface Soil	Subsurface Soil	Mean	Unit	Depth	Unit × Depth
**V**	5.39 ± 1.01 b	4.29 ± 0.76 b	4.84 B	25.1 ± 7.6 a	19.6 ± 7.1 a	22.4 A	31.51 **	1.10 ns	0.49 ns
**Cr**	18.8 ± 5.7 a	11.0 ± 1.5 b	14.9 A	13.9 ± 2.0 ab	11.4 ± 2.6 b	12.7 A	1.51 ns	11.9 *	2.08 ns
**Mn**	2842 ± 3378 a	157 ± 145 b	1500 A	354 ± 316 b	110 ± 66 b	232 B	4.83 *	16.17 **	3.74 ns
**Co**	14.5 ± 16.4 a	1.47 ± 0.33 b	8.03 A	4.15 ± 2.59 b	1.83 ± 0.63 b	2.99 A	1.79 ns	17.57 **	3.56 ns
**Ni**	48.5 ± 48.2 a	10.6 ± 0.5 bc	29.5 A	18.6 ± 4.3 b	10.6 ± 2.6 c	14.6 A	3.17 ns	20.65 **	2.70 ns
**Cu**	217 ± 191 a	12.0 ± 8.3 a	114 A	70.4 ± 39.4 a	34.5 ± 31.6 a	52.5 A	0.34 ns	1.85 ns	0.03 ns
**As**	20.0 ± 2.9 c	14.9 ± 3.6 c	17.5 B	108 ± 20 a	72.3 ± 15.6 b	90.4 A	86.43 **	6.95 *	3.91 ns
**Se**	3.54 ± 3.06 ab	2.32 ± 2.01 ab	2.93 A	3.83 ± 0.73 a	1.61 ± 2.07 b	2.72 A	0.05 ns	3.55 ns	0.30 ns
**Sr**	596 ± 138 bc	494 ± 192 c	545 B	1147 ± 339 a	986 ± 430 ab	1066 A	10.24 *	0.65 ns	0.03 ns
**Y**	13.7 ± 13.2 a	3.84 ± 2.5 bc	8.18 A	8.84 ± 4.78 ab	2.56 ± 1.3 c	5.70 A	1.14 ns	14.40 *	0.02 ns
**Zr**	2.54 ± 1.34 a	4.16 ± 4.18 a	3.35 A	4.27 ± 1.65 a	2.30 ± 1.11 a	3.28 A	0.01 ns	0.03 ns	3.47 ns
**Mo**	10.3 ± 1.9 b	12.11 ± 4.24 b	11.2 B	16.1 ± 1.8 b	22.8 ± 8.5 a	19.5 A	9.08 *	2.40 ns	0.83 ns
**Cd**	10.2 ± 7.2 a	3.84 ± 1.05 a	7.05 A	9.75 ± 8.12 a	2.08 ± 1.89 a	5.91 A	1.18 ns	1.74 ns	0.99 ns
**In**	0.05 ± 0.43 a	nd	0.25 A	0.13 ± 0.24 b	0.02 ± 0.07 b	0.08 A	2.51 ns	8.24 *	3.40 ns
**Sn**	nd	nd	ndA	0.07 ± 0.18	nd	0.03 A	0.40 ns	0.40 ns	0.40 ns
**Sb**	30.8 ± 6.2 b	25.6 ± 9.2 b	28.2 B	108 ± 41 a	141 ± 75 a	125 A	58.13 *	0 ns	1.37 ns
**La**	2.65 ± 0.52 b	2.59 ± 1.63 b	2.62 B	10.7 ± 5.4 a	4.0 ± 3.04 b	7.38 A	6.41 *	3.30 ns	3.18 ns
**Ce**	7.96 ± 3.59 b	5.58 ± 3.8 b	6.77 B	23.4 ± 11.6 a	8.97 ± 6.16 b	16.2 A	5.46 *	4.36 ns	2.25 ns
**Pr**	1.91 ± 1.79 ab	0.62 ± 0.64 b	1.26 A	3.44 ± 1.69 a	1.15 ± 0.88 b	2.30 A	2.45 ns	7.34 *	0.58 ns
**Bi**	nd	nd	nd	nd	nd	nd	-	-	-
**Th**	1.54 ± 0.98 a	2.08 ± 1.54 a	1.81 A	2.92 ± 1.17 a	1.67 ± 1.72 a	2.29 A	0.48 ns	0.27 ns	1.64 ns
**Ge**	0.58 ± 0.54 ab	ndb	0.29 A	1.96 ± 1.81 a	0.49 ± 0.81 b	1.23 A	2.44 ns	2.92 ns	0.53 ns
**Rb**	10.2 ± 2.9 b	10.7 ± 2.4 b	10.4 B	29.0 ± 12.7 a	16.7 ± 7.9 b	22.8 A	7.45 *	1.68 ns	1.99 ns
**Cs**	0.52 ± 0.68 b	0.10 ± 0.17 b	0.31 B	2.77 ± 1.32 a	2.49 ± 1.43 a	2.63 A	15.19 *	0.35 ns	0.01 ns
**U**	6.4 ± 3.77 a	2.72 ± 2.63 a	4.56 A	4.46 ± 0.86 a	3.72 ± 2.79 a	4.09 A	0.16 ns	3.50 ns	1.54 ns
**Fe**	2418 ± 1207 ab	1665 ± 2171 ab	2042 A	2870 ± 1742 a	1140 ± 703 b	2005 A	0 ns	3.09 ns	0.48 ns
**Pb**	850 ± 603 a	324 ± 94 a	587 A	2209 ± 2051 a	629 ± 528 a	1419 A	0.71 ns	3.83 ns	0.20 ns
**Zn**	2231 ± 2361 a	434 ± 298 bc	1333 A	951 ± 731 ab	344 ± 198 c	648 A	1.02 ns	9.55 *	0.58 ns
**Al**	2000 ± 2645 a	666 ± 1154 a	1333 A	714 ± 951 a	495 ± 763 a	604 A	0.1 ns	0.43 ns	0.39 ns

Nd non-detected. Different letters indicate significant differences (*p* < 0.05) between means after an ANOVA test. * *p* < 0.05; ** *p* < 0.01; ns: non-significant (*p* > 0.05).

**Table 5:** Metal(loid)s and REEs concentration in different plants.

		V			Cr			Mn *			Co		
		Root	Shoot	Mean	Root	Shoot	Mean	Root	Shoot	Mean	Root	Shoot	Mean
Unit S1	*S. o.*	2263 ± 2530 ab	849 ± 256 bcd	1556 AB	976 ± 317 c	1610 ± 550 abc	1293 A	124 ± 36 ab	220 ± 42 a	172 A	659 ± 109 a	439 ± 155 abc	549 A
	*S. t.*	964 ± 230 bc	729 ± 324 bcde	846 BC	807 ± 69 c	3654 ± 1511 ab	2231 A	22.3 ± 2.6 def	52.5 ± 2.1 bcd	37.4 C	201 ± 49 c–f	157 ± 55 d–h	179 BC
	*P. m.*	2489 ± 687 a	1036 ± 164 abc	1762 A	5030 ± 6660 abc	6617 ± 4636 a	5824 A	68.4 ± 55.9 bcd	24.3 ± 6.9 de	46.4 C	424 ± 426 abc	213 ± 37 b–f	318 B
Unit S2	*S. o.*	530 ± 212 cde	399 ± 158 de	464 C	1212 ± 923 c	1301 ± 587 bc	1256 A	70.1 ± 36.9 bc	130 ± 15 b	100 B	176 ± 128 d–h	129 ± 77 e–h	153 C
	*S. t.*	199 ± 1264 ab	293 ± 99 ef	1142 BC	1783 ± 831 abc	3633 ± 1272 ab	2708 A	30.9 ± 19.1 cde	23.0 ± 5.7 def	26.9 C	283 ± 130 b–e	74.7 ± 15 hi	179 C
	*P. m.*	1855 ± 1272 ab	138 ± 47 f	997 C	9184 ± 11,567 a	1449 ± 302 bc	5317 A	19.8 ± 20.9 ef	8.9 ± 1.3 f	14.4 E	493 ± 205 ab	48.3 ± 17 i	270 BC
	*A. h.*	607 ± 285 cde	622 ± 99 cde	614 C	1437 ± 366 bc	1037 ± 190 bc	1237 A	17.1 ± 6.3 ef	20.8 ± 5.7 ef	18.9 DE	101 ± 45 ghi	104 ± 5 fgh	102 C
		Ni			Cu *			As *			Se		
		Root	Shoot	Mean	Root	Shoot	Mean	Root	Shoot	Mean	Root	Shoot	Mean
Unit S1	*S. o.*	2278 ± 708 ab	2512 ± 782 ab	2395 A	10.6 ± 6.08 abc	15.5 ± 5.1 a	13.08 A	2.11 ± 1.5 bcd	0.60 ± 0.2 ef	1.35 BC	101 ± 64 ab	89.4 ± 12 ab	95.3 A
	*S. t.*	704 ± 82 bcd	918 ± 319 a–d	811 A	6.8 ± 4.5 bcd	3.54 ± 0.53 def	5.22 BC	2.44 ± 0.6 bc	0.60 ± 0.1 def	1.52 B	41.6 ± 17 b–e	40.5 ± 15 b–e	41.1 B
	*P. m.*	2124 ± 1676 abc	2578 ± 1610 a	2351 A	12.13 ± 6.6 ab	2.95 ± 0.66 efg	7.54 B	2.37 ± 1 a	1.39 ± 0.2 be	1.25 A	99.3 ± 39 a	58.7 ± 4 a–e	79.1 A
Unit S2	*S. o.*	922 ± 533 a–d	945 ± 493 abcd	933 A	3.77 ± 0.99 def	5.60 ± 1.27 cde	4.68 BC	0.95 ± 0.40 cde	0.34 ± 0.1 fg	0.65 C	34.3 ± 1 cde	45.7 ± 19 a–e	40.0 B
	*S. t.*	1039 ± 407 a–d	6571 ± 1564 ab	3805 A	4.01 ± 1.73 def	2.56 ± 0.37 fg	3.28 CD	4.44 ± 3.6 b	0.42 ± 0.1 fg	2.43 B	89.8 ± 72 abc	39.9 ± 10 b–e	64.9 B
	*P. m.*	2921 ± 1620 a	393 ± 53 d	1657 A	4.44 ± 2.08 def	1.86 ± 0.57 g	3.15 D	3.20 ± 2.7 bc	0.19 ± 0.03 g	1.70 BC	76.8 ± 53 a–e	26.3 ± 11 e	51.6 B
	*A. h.*	674 ± 356 cd	781 ± 115 abcd	728 A	10.48 ± 5.18 abc	9.05 ± 2.38 abc	9.77 A	0.33 ± 0.77 c–e	0.55 ± 0.07 ef	0.94 BC	27.6 ± 9 de	67 ± 41 abc	47.3 B
		Sr *			Y			Zr			Mo		
		Root	Shoot	Mean	Root	Shoot	Mean	Root	Shoot	Mean	Root	Shoot	Mean
Unit S1	*S. o.*	72.4 ± 41.8 bc	157 ± 8 a	115 A	1160 ± 1057 ab	519 ± 27 bcd	839 AB	200 ± 182 ab	100 ± 51 a–d	150 AB	290 ± 218 cd	521 ± 523 bcd	405 CD
	*S. t.*	5.9 ± 0.51 f	6.43 ± 1.22 f	6.19 D	946 ± 624 abc	236 ± 73 de	591 BC	30.7 ± 0.7 ef	56.7 ± 9 de	43.7 D	53.1 ± 20 e	76.6 ± 2 e	64.8 E
	*P. m.*	44.2 ± 42.22 cd	13.8 ± 1.6 e	29.0 C	2186 ± 1189 a	517 ± 92 bcd	1352 A	100 ± 73 bcd	68.4 ± 4 cde	84.4 BC	179 ± 18 d	232 ± 43 cd	206 D
Unit S2	*S. o.*	120 ± 3 ab	171 ± 11 a	145 A	248 ± 95 de	146 ± 97 e	206 DE	132 ± 22 abc	95.8 ± 45 a–d	114 AB	191 ± 71 d	1289 ± 586 a	740 BC
	*S. t.*	46.4 ± 26.3 c	15.1 ± 1.4 e	30.8 C	926 ± 629 abc	122 ± 27 ef	524 CD	188 ± 82 ab	54.3 ± 32 de	121 BC	442 ± 44 bc	653 ± 193 ab	548 AB
	*P. m.*	48.0 ± 21.5 c	17.6 ± 4.4 de	32.8 C	527 ± 561 cde	47.6 ± 24 f	286 E	169 ± 86 abc	21.7 ± 5 f	95.6 CD	792 ± 183 ab	1138 ± 503 a	965 A
	*A. h.*	61.7 ± 16.5 bc	45.0 ± 3.7 c	53.4 B	191 ± 91 de	203 ± 25 de	197 DE	191 ± 79 ab	198 ± 29 a	194 A	661 ± 124 ab	665 ± 221 ab	663 AB
		Cd			In			Sn			Sb		
		Root	Shoot	Mean	Root	Shoot	Mean	Root	Shoot	Mean	Root	Shoot	Mean
Unit S1	*S. o.*	1290 ± 501 bc	3487 ± 2788 b	2389 A	25.4 ± 16 bc	12.2 ± 9 cde	18.8 B	104 ± 37 bcd	111 ± 28 bc	108 BC	309 ± 291 cde	157 ± 44 ef	233 C
	*S. t.*	164 ± 55 de	71 ± 4 ef	117 C	27.5 ± 7 b	7.6 ± 3 ef	17.6 BC	157 ± 17 ab	96.4 ± 16 bcd	126 AB	64.2 ± 42 gh	58.6 ± 21 gh	61.4 D
	*P. m.*	17,694 ± 1803 a	240 ± 117 de	8967 A	573 ± 272 a	28.7 ± 6 b	301.2 A	280 ± 144 a	127 ± 17 bc	204 A	2528 ± 207 a	256 ± 28 cde	1392 A
Unit S2	*S. o.*	1662 ± 1336 bc	2931 ± 3735 bc	2297 A	8.2 ± 3 ef	12.5 ± 4 b–e	10.3 BC	108 ± 34 bcd	88.9 ± 40 cd	98.8 CD	626 ± 292 bc	132 ± 85 efg	379 BC
	*S. t.*	813 ± 972 cd	213 ± 165 de	513 B	20.4 ± 16 be	3.6 ± 0.6 f	12.1 CD	119 ± 68 bcd	58.6 ± 11 de	88.9 CD	548 ± 270 bcd	72.9 ± 32 fgh	310 C
	*P. m.*	260 ± 313 de	26.5 ± 2 f	143 C	21.2 ± 12 bcd	1 ± 0.1 g	11.1 D	112 ± 58 bcd	38.5 ± 8 e	75.3 D	1170 ± 725 ab	41.7 ± 18 h	606 C
	*A. h.*	1784 ± 397 bc	1032 ± 397 bc	1408 A	13.3 ± 12 cde	7.2 ± 1 ef	10.2 BC	106 ± 50 bcd	135 ± 6 abc	120 AC	981 ± 593 ab	203 ± 33 de	592 AB
		La			Ce			Pr			Bi		
		Root	Shoot	Mean	Root	Shoot	Mean	Root	Shoot	Mean	Root	Shoot	Mean
Unit S1	*S. o.*	701 ± 570 bcd	497 ± 48 bcd	599 B	979 ± 886 cde	519 ± 120 cde	749 BC	201 ± 154 bc	110.2 ± 12 dc	156 BC	29.5 ± 22 ab	9.2 ± 6 cde	19.3 BC
	*S. t.*	479 ± 129 bcd	375 ± 124 cd	427 BC	1073 ± 475 bcd	542 ± 186 cde	807 BC	209 ± 105 bc	92.9 ± 28 cd	151 BC	80.2 ± 90 a	12.2 ± 1 b–e	46.2 AB
	*P. m.*	2958 ± 2307 a	734 ± 69 bc	1846 A	4145 ± 3072 a	1307 ± 190 abc	2726 A	744 ± 501 a	198 ± 27 bc	471 A	55.7 ± 14 a	21.4 ± 4 abc	38.5 A
Unit S2	*S. o.*	451 ± 122 cd	255 ± 128 de	353 BC	596 ± 263 cde	329 ± 162 ef	463 C	125 ± 47 dc	58.8 ± 30 de	92.1 BC	6.8 ± 4 de	9.7 ± 5 cde	8.3 C
	*S. t.*	1852 ± 1850 ab	272 ± 84 de	1062 B	4409 ± 4982 ab	461 ± 124 cde	2435 AB	706 ± 764 ab	69.6 ± 21 cde	388 AB	11.1 ± 4 b–e	6.4 ± 1 de	8.7 C
	*P. m.*	678 ± 545 bcd	112 ± 46 e	395 C	1426 ± 1089 bcd	161 ± 71 f	793 C	337 ± 279 bc	26.9 ± 11 e	182 C	7.2 ± 6 e	0 f	3.6 D
	*A. h.*	270 ± 148 de	369 ± 70 cd	320 BC	403 ± 170 def	435 ± 56 def	419 C	80.2 ± 42 cde	83 ± 17 cd	81.6 C	6.2 ± 3 de	14.9 ± 3 bcd	10.6 C
		Th			Ge			Rb *			Cs		
		Root	Shoot	Mean	Root	Shoot	Mean	Root	Shoot	Mean	Root	Shoot	Mean
Unit S1	*S. o.*	131 ± 131 de	41.4 ± 18 ef	86.3 C	803 ± 539 a	133 ± 9 bc	468 A	7.58 ± 2.55 bc	38.7 ± 13.4 a	23.1 A	362 ± 126 def	3331 ± 1917 ab	1847 B
	*S. t.*	88.2 ± 28 def	80.7 ± 22 def	84.5 C	74.9 ± 19 cd	36.1 ± 10 de	55.6 C	2.31 ± 0.96 e	2.04 ± 1.1 ef	2.17 BC	512 ± 271 cde	398 ± 230 def	455 CD
	*P. m.*	657 ± 559 a	186 ± 37 bcd	421.9 A	276 ± 64 ab	261.7 ± 72 b	268.9 A	2.98 ± 0.69 cde	1.38 ± 0.4 ef	2.1 BC	763 ± 406 cd	334 ± 88 def	549 BC
Unit S2	*S. o.*	301 ± 134 abc	50.1 ± 40 ef	176 BC	61.1 ± 16 cde	25.6 ± 11 e	43.4 C	1.18 ± 0.64 b	64.1 ± 32.2 a	38.0 A	1782 ± 1677 bc	10,748 ± 7663 a	6265 A
	*S. t.*	677 ± 609 ab	84 ± 27 def	380 AB	231 ± 109 b	71.6 ± 29 cd	151 B	3.51 ± 0.31 de	0.81 ± 0.3 f	2.1 C	644 ± 543 cde	273 ± 223 ef	458 CD
	*P. m.*	274 ± 186 a–d	32.5 ± 11 f	153 C	186 ± 181 bc	64.7 ± 76 de	125 BC	3.34 ± 2.84 e	1.95 ± 0.3 ef	2.6 BC	354 ± 185 def	117 ± 8 f	235 D
	*A. h.*	141 ± 57 cd	109 ± 29 cde	125 BC	63.6 ± 32 cde	31.2 ± 4 de	47.4 C	1.79 ± 0.43 ef	6.42 ± 2.6 bcd	4.1 B	255 ± 94 def	320 ± 282 def	287 CD
		U			Pb *			Zn *			Al *		
		Root	Shoot	Mean	Root	Shoot	Mean	Root	Shoot	Mean	Root	Shoot	Mean
Unit S1	*S. o.*	256.6 ± 201 abc	61.7 ± 15 cde	159.2 A	46.7 ± 28 b–f	31.4 ± 6 c–f	39.1 B	236.9 ± 121 a	547 ± 339 a	402.2 A	49.3 ± 39 f	33.7 ± 19 f	41.5 E
	*S. t.*	949.8 ± 799 a	69.8 ± 9 cde	509.8 A	92.6 ± 7 bc	4.3 ± 1 g	48.5 B	24.6 ± 4 de	26.0 ± 6.2 de	25.3 CD	750 ± 175 a	407 ± 22 abc	578 A
	*P. m.*	861.1 ± 443 a	137.5 ± 34 bcd	499.3 A	1943.6 ± 694 a	31.1 ± 17 def	987.4 A	687.3 ± 484 a	92.4 ± 29.3 b	389 A	60.9 ± 29 ef	171 ± 50 cd	116 D
Unit S2	*S. o.*	488.6 ± 249 ab	52.5 ± 23 de	270.6 A	51.4 ± 20 b–e	16.8 ± 6 f	34.1 B	60.9 ± 27 bcd	84.9 ± 43 bc	72.9 B	242 ± 76 bcd	108 ± 28 de	175 CD
	*S. t.*	2437.1 ± 3997 ab	43.8 ± 7 de	1240.5 A	162.7 ± 164 b	14.2 ± 1 f	88.5 B	55.8 ± 32 bcd	36.2 ± 13.9 cd	46.0 BC	311 ± 225 bcd	240 ± 187 cd	275 BC
	*P. m.*	451.8 ± 382 ab	27.1 ± 7 e	239.5 A	55.3 ± 61 c–f	1.8 ± 0.2 g	28.5 C	49.2 ± 64 de	12.5 ± 3.8 e	30.8 D	660 ± 506 ab	236 ± 121 bcd	448 AB
	*A. h.*	345 ± 204 ab	64.5 ± 22 cde	204.8 A	112.6 ± 144 bcd	17.2 ± 4 ef	64.9 B	60.5 ± 22.4 bcd	53.3 ± 10.9 bcd	56.9 B	163 ± 43 cd	237 ± 63 bcd	200 BC
		Fe *											
		Root	Shoot	Mean									
Unit S1	*S. o.*	159 ± 124 bcd	238 ± 43 abc	199 B									
	*S. t.*	131 ± 30 bcd	146 ± 45 bcd	135 BC									
	*P. m.*	495 ± 309 a	261 ± 40 abc	378 A									
Unit S2	*S. o.*	206 ± 83 bcd	138 ± 67 cd	172 BC									
	*S. t.*	283 ± 109 ab	96.3 ± 33 d	190 BC									
	*P. m.*	295 ± 137 ab	37.9 ± 8 e	166 C									
	*A. h.*	170 ± 86 bcd	251 ± 48 abc	210 B									

* Concentration of Mn, As, Sr, Cu, Rb, Pb, Zn, Al, and Fe are represented as mg kg^−1^, and other elements are represented as µg kg^−1^. Different letters indicate significant differences (*p* < 0.05) between means after an ANOVA test. *S.o.: S. oppositifolia*, *S. t.*: *S. tenacissima*, *P. m.*: *P. miliaceum*, *A. h.*: *A. herba-alba*.

**Table 6:** Bioaccumulation Factor (BCF) for different metal(loid)s and REEs in Unit S1 and Unit S2.

	BCF_root_							BCF_shoot_						
	Unit 1			Unit 2				Unit 1			Unit 2			
	*S.o.*	*S. t.*	*P. m.*	*S.o.*	*S. t.*	*P. m.*	*A. h.*	*S.o.*	*S. t.*	*P. m.*	*S.o.*	*S. t.*	*P. m.*	*A. h.*
**V**	95	41	90	3.4	13	12	3.9	31	31	42	2.6	1.9	0.9	4
**Cr**	30	20	226	6.4	9.4	48	7.5	51	79	218	6.8	19	7.6	5.4
**Mn**	1.9	0.3	0.9	1	0.4	0.3	0.2	3.4	0.8	0.4	1.9	0.3	0.1	0.3
**Co**	1.5	0.4	0.9	0.2	0.4	0.7	0.1	1	0.4	0.5	0.2	0.1	0.1	0.1
**Ni**	3.8	1.2	4.1	1.1	1.3	3.6	0.8	4.6	1.5	5.1	1.2	8.1	0.5	1
**Cu**	1.8	2	2.6	0.4	0.4	0.5	1.1	4	0.7	0.6	0.6	0.3	0.2	0.9
**As**	69	81	790	6.8	32	23	9.5	19	21	47	2.5	3	1.4	3.9
**Se**	2.1	0.9	2.4	1.3	3.4	2.9	1	1.9	0.8	1.3	1.7	1.5	1	2.5
**Sr**	4.3	0.4	2.9	4.6	1.8	1.8	2.3	9.4	0.4	0.8	6.5	0.6	0.7	1.7
**Y**	0.5	0.5	1.2	0.4	1.6	0.9	0.3	0.3	0.1	0.3	0.3	0.2	0.1	0.4
**Zr**	15	2.6	11	7.4	10	9.4	11	8.3	4.6	5.8	5.3	3	1.2	11
**Mo**	12	2.4	7.5	5.5	13	23	19	22	3.2	10	37	19	33	19
**Cd**	0.3	0	3	1.2	0.6	0.2	1.2	0.7	0	0.1	2	0.1	0	0.7
**In**	5.8	8.2	125	2.2	5.4	5.6	3.5	2.4	2	8.3	3.3	1	0.3	1.9
**Sb**	3.2	0.6	25	1.9	1.7	3.6	3	1.6	0.7	3	0.4	0.2	0.1	0.6
**La**	1.7	1.5	9.9	1.2	5.1	1.9	0.7	1.5	1	2.2	0.7	0.7	0.3	1
**Ce**	1	1.2	4.3	0.4	3.1	1	0.3	0.5	0.6	1.4	0.2	0.3	0.1	0.3
**Pr**	1.5	1.9	7.6	1	5.8	2.8	0.7	1	0.8	1.8	0.5	0.6	0.2	0.7
**Pb**	0.1	0.2	3.7	0.1	0.2	0.1	0.2	0.1	0	0.1	0	0	0	0
**Bi**	1.1	3	2.4	0.2	0.4	0.3	0.2	0.4	0.5	0.9	0.3	0.2	0	0.5
**Th**	3	2.4	16	4.2	9.3	3.8	1.9	1	2.1	5.5	0.7	1.2	0.4	1.5
**Ge**	20	1.8	6.7	0.7	2.5	2	0.7	3.3	0.9	6.3	0.3	0.8	0.7	0.3
**Cs**	41	38	76	128	46	25	18	372	34	29	770	20	8.4	23
**U**	24	157	85	28	139	26	20	6.3	7.8	18	3	2.5	1.5	3.7
**Zn**	2.9	0.3	7.6	0.6	0.5	0.5	0.6	6.5	0.3	1.1	0.8	0.3	0.1	0.5
**Al**	4.7	112	7.1	40	52	110	27	3.5	55	20	18	40	39	39
**Fe**	9.6	8.4	33	16	22	22	13	15	9	17	11	7.3	2.9	19

*S. o.*: *S. oppositifolia*, *S. t.*: *S. tenacissima*, *P. m.*: *P. miliaceum*, *A. h.*: *A. herba-alba*.

**Table 7:** Reference concentrations of 27 elements.

	Background Level (mg kg^−1^)		Soil Quality Reference Values (mg kg^−1^)		
	Sanchez et al. [53]	Martínez-Martínez [87]	IGME [54]		Ballesta et al. [56]
**Zn**	55.2	48	-	**Mo**	2.0
**Fe**	-	-	2.02	**Rb**	234
**Al**	-	-	4540	**Cs**	14.2
**Pb**	9.8	-	-	**V**	123
**Mn**	664	-	359	**Sr**	1868
**Cd**	0.12	0.13	-	**Zr**	413
**Co**	7.7	24.4	-	**Sn**	8.7
**Ni**	16.8	-	-	**Y**	38.3
**Cr**	44.6	115	-	**La**	48.4
**Cu**	18.7	43.6	-	**Ce**	97.9
**As**	8.1	-	14	**Bi**	2.2
**Se**	0.22	-	0.7	**Ge**	1.3
**Sb**	1.1	-	1	**U**	10.3
**Th**	0.1	-	-		

## Data Availability

The data is contained within the manuscript and Supplementary Materials.

## Supplementary Materials

The following supporting information can be downloaded at: https://www.mdpi.com/article/10.3390/plants12061219/s1, Table S1: Linear correlation matrix of pH, EC, sand, silt, clay and metal(loid)s/REEs in all soil samples (n = 20); Table S2: Linear correlation matrix of total metal(loid)s and REEs in all soil samples (n = 20).

## References

[1] Tchounwou P.B., Yedjou C.G., Patlolla A.K., Sutton D.J. (2012). Heavy metal toxicity and the environment. Exp. Suppl..

[2] Ramírez O., Sánchez de la Campa A.M., Sánchez-Rodas D., de la Rosa J.D. (2020). Hazardous trace elements in thoracic fraction of airborne particulate matter: Assessment of temporal variations, sources, and health risks in a megacity. Sci. Total. Environ..

[3] Pagano G., Aliberti F., Guida M., Oral R., Siciliano A., Trifuoggi M., Tommasi F. (2015). Rare earth elements in human and animal health: State of art and research priorities. Environ. Res..

[4] Zhang M., Zhang T., Zhou L., Lou W., Zeng W., Liu T., Yin H., Liu H., Liu X., Mathivanan K. (2022). Soil microbial community assembly model in response to heavy metal pollution. Environ. Res..

[5] Zhang W., Long J., Wei Z., Alakangas L. (2016). Vertical distribution and historical loss estimation of heavy metals in an abandoned tailings pond at HTM copper mine, northeastern China. Environ. Earth Sci..

[6] Schaider L.A., Senn D.B., Estes E.R., Brabander D.J., Shine J.P. (2014). Sources and fates of heavy metals in a mining-impacted stream: Temporal variability and the role of iron oxides. Sci. Total. Environ..

[7] Wang P., Sun Z., Hu Y., Cheng H. (2019). Leaching of heavy metals from abandoned mine tailings brought by precipitation and the associated environmental impact. Sci. Total. Environ..

[8] Ding Q., Cheng G., Wang Y., Zhuang D. (2017). Effects of natural factors on the spatial distribution of heavy metals in soils surrounding mining regions. Sci. Total. Environ..

[9] Yun S.-W., Kang D.-H., Ji W.-H., Jung M.-H., Yu C. (2020). Distinct Dispersion of As, Cd, Pb, and Zn in Farmland Soils near Abandoned Mine Tailings: Field Observation Results in South Korea. J. Chem..

[10] Acosta J.A., Jansen B., Kalbitz K., Faz A., Martínez-Martínez S. (2011). Salinity increases mobility of heavy metals in soils. Chemosphere.

[11] Dimirkou A., Ioannou Z., Golia E.E., Danalatos N., Mitsios I.K. (2003). Sorption of Cadmium and Arsenic by Goethite and Clinoptilolite. Commun. Soil Sci. Plant Anal..

[12] Yang L., Zhou M.L., Lu R.N., Liao Y.P.V. (2012). Tourism Development and Exploration of Abandoned Mine—Taking National Mine Park of Jianghe Coal Mine in Chongqing as an Example. Adv. Mater. Res..

[13] Pascaud G., Leveque T., Soubrand M., Boussen S., Joussein E., Dumat C. (2013). Environmental and health risk assessment of Pb, Zn, As and Sb in soccer field soils and sediments from mine tailings: Solid speciation and bioaccessibility. Environ. Sci. Pollut. Res..

[14] Colín-Torres C.G., Murillo-Jiménez J.M., Del Razo L.M., Sánchez-Peña L.C., Becerra-Rueda O.F., Marmolejo-Rodríguez A.J. (2014). Urinary arsenic levels influenced by abandoned mine tailings in the Southernmost Baja California Peninsula, Mexico. Environ. Geochem. Health.

[15] Peñalver-Alcalá A., Álvarez-Rogel J., Peixoto S., Silva I., Silva A.R.R., González-Alcaraz M.N. (2021). The relationships between functional and physicochemical soil parameters in metal(loid) mine tailings from Mediterranean semiarid areas support the value of spontaneous vegetation colonization for phytomanagement. Ecol. Eng..

[16] Anoopkumar A.N., Rebello S., Devassy E., Kavya Raj K., Puthur S., Aneesh E.M., Sindhu R., Binod P., Pandey A., Inamuddin A.M.I., Lichtfouse E., Asiri A.M. (2020). Phytoextraction of Heavy Metals. Methods for Bioremediation of Water and Wastewater Pollution.

[17] Shackira A.M., Puthur J.T., Srivastava S., Srivastava A.K., Suprasanna P. (2019). Phytostabilization of Heavy Metals: Understanding of Principles and Practices. Plant-Metal Interactions.

[18] Martínez-López S., Martínez-Sánchez M.J., Pérez-Sirvent C., Bech J., del Carmen Gómez Martínez M., García-Fernandez A.J. (2014). Screening of wild plants for use in the phytoremediation of mining-influenced soils containing arsenic in semiarid environments. J. Soils Sediments.

[19] Kołodziej B., Antonkiewicz J., Bielińska E.J., Witkowicz R., Dubis B. (2023). Recovery of microelements from municipal sewage sludge by reed canary grass and giant miscanthus. Int. J. Phytoremediation.

[20] Tarla D.N., Erickson L.E., Hettiarachchi G.M., Amadi S.I., Galkaduwa M., Davis L.C., Nurzhanova A., Pidlisnyuk V. (2022). Remediation of soils on municipal rendering plant territories using Mscanthus x giganteus. Environ. Sci. Pollut. Res..

[21] Martínez-Carlos J., Martínez-Martínez S., Faz A., Zornoza R., Gabarrón M., Soriano-Disla M., Gómez-López M.D., Acosta J.A. (2021). Are the soils and vegetation of a forest close to tailings ponds affected by metals and arsenic?. Environ. Geochem. Health.

[22] Suo Y., Tang N., Li H., Corti G., Jiang L., Huang Z., Zhang Z., Huang J., Wu Z., Feng C. (2021). Long-term effects of phytoextraction by a poplar clone on the concentration, fractionation, and transportation of heavy metals in mine tailings. Environ. Sci. Pollut. Res..

[23] Zhu H., Cheng R., Bañuelos G., Centofanti T. (2019). Feasibility of growing halophyte “agretti” (Salsola soda) as an alternative boron-tolerant food crop in unproductive boron-laden regions. Plant Soil.

[24] Toderich K.N., Shuyskaya E.V., Khujanazarov T.M., Ismail S., Kawabata Y., Ashraf M., Ozturk M., Ahmad  M.S.A. (2010). The Structural and Functional Characteristics of Asiatic Desert Halophytes for Phytostabilization of Polluted Sites. Plant Adaptation and Phytoremediation.

[25] Heckenroth A., Rabier J., Dutoit T., Torre F., Prudent P., Laffont-Schwob I. (2016). Selection of native plants with phytoremediation potential for highly contaminated Mediterranean soil restoration: Tools for a non-destructive and integrative approach. J. Environ. Manag..

[26] Parraga-Aguado I., Querejeta J.-I., González-Alcaraz M.-N., Jiménez-Cárceles F.J., Conesa H.M. (2014). Usefulness of pioneer vegetation for the phytomanagement of metal(loid)s enriched tailings: Grasses vs. shrubs vs. trees. J. Environ. Manag..

[27] Azizi M., Faz A., Zornoza R., Martínez-Martínez S., Shahrokh V., Acosta J.A. (2022). Environmental pollution and depth distribution of metal(loid)s and rare earth elements in mine tailing. J. Environ. Chem. Eng..

[28] Gabarrón M., Faz A., Martínez-Martínez S., Acosta J.A. (2018). Change in metals and arsenic distribution in soil and their bioavailability beside old tailing ponds. J. Environ. Manag..

[29] Cobertera E. (1993). Edafología Aplicada.

[30] Porta J., López-Acevedo M., Roquero C. (1999). Edafología Para la Agricultura y el Medio Ambiente.

[31] IUSS Working Group WRB (2022). World Reference Base for Soil Resources. International Soil Classification System for Naming Soils and Creating Legends for Soil Maps.

[32] Tuo D.F., Xu M.X., Ma X.X., Zheng S.Q. (2014). Impact of wind-water alternate erosion on the characteristics of sediment particles. Ying Yong Sheng Tai Xue Bao J. Appl. Ecol..

[33] Midhat L., Ouazzani N., Hejjaj A., Ouhammou A., Mandi L. (2019). Accumulation of heavy metals in metallophytes from three mining sites (Southern Centre Morocco) and evaluation of their phytoremediation potential. Ecotoxicol. Environ. Saf..

[34] Zhao X.Q., Shen R.F. (2018). Aluminum–Nitrogen Interactions in the Soil–Plant System. Front. Plant Sci..

[35] Balintova M., Petrilakova A. (2011). Study of pH Influence on Selective Precipitation of Heavy Metals from Acid Mine Drainage. Chem. Eng. Trans..

[36] Alloway B. (2013). Sources of Heavy Metals and Metalloids in Soils.

[37] Gilkes R.J., McKenzie R.M., Graham R.D., Hannam R.J., Uren N.C. (1988). Geochemistry and Mineralogy of Manganese in Soils. Manganese in Soils and Plants: Proceedings of the International Symposium on ‘Manganese in Soils and Plants’ held at the Waite Agricultural Research Institute, The University of Adelaide, Glen Osmond, Australia, 22–26 August 1988.

[38] Queiroz H.M., Ying S.C., Abernathy M., Barcellos D., Gabriel F.A., Otero X.L., Nóbrega G.N., Bernardino A.F., Ferreira T.O. (2021). Manganese: The overlooked contaminant in the world largest mine tailings dam collapse. Environ. Int..

[39] Martínez-Pagán P., Faz A., Acosta J., Carmona D.M., Martínez-Martínez S. (2011). A multidisciplinary study for mining landscape reclamation: A study case on two tailing ponds in the Region of Murcia (SE Spain). Phys. Chem. Earth.

[40] Kabata-Pendias A., Mukherjee A.B. (2007). Trace Elements from Soil to Human.

[41] Ahmady-Birgani H., Engelbrecht J.P., Bazgir M. (2019). How different source regions across the Middle East change aerosol and dust particle characteristics. Desert.

[42] Edahbi M., Plante B., Benzaazoua M., Ward M., Pelletier M. (2018). Mobility of rare earth elements in mine drainage: Influence of iron oxides, carbonates, and phosphates. Chemosphere.

[43] Roth E., Bank T., Howard B., Granite E. (2017). Rare Earth Elements in Alberta Oil Sand Process Streams. Energy Fuels.

[44] Balboni E., Simonetti A., Spano T., Cook N.D., Burns P.C. (2017). Rare-earth element fractionation in uranium ore and its U(VI) alteration minerals. Appl. Geochem..

[45] Ayora C., Macías F., Torres E., Lozano A., Carrero S., Nieto J.-M., Pérez-López R., Fernández-Martínez A., Castillo-Michel H. (2016). Recovery of rare earth elements and yttrium from passive-remediation systems of acid mine drainage. Environ. Sci. Technol..

[46] Pereira W.V.d.S., Ramos S.J., Melo L.o.C.A., Braz A.M.d.S., Dias Y.N., Almeida G.V.d., Fernandes A.R. (2022). Levels and environmental risks of rare earth elements in a gold mining area in the Amazon. Environ. Res..

[47] Tao Y., Shen L., Feng C., Yang R., Qu J., Ju H., Zhang Y. (2022). Distribution of rare earth elements (REEs) and their roles in plant growth: A review. Environ. Pollut..

[48] Mleczek P., Borowiak K., Budka A., Niedzielski P. (2018). Relationship between concentration of rare earth elements in soil and their distribution in plants growing near a frequented road. Environ. Sci. Pollut. Res..

[49] Cabral A.R., Lehmann B., Kwitko R., Costa C.H.C. (2002). The Serra Pelada Au-Pd-Pt deposit, Carajas Mineral Province, Northern Brazil: Reconnaissance mineralogy and chemistry of very high grade palladian gold mineralization. Econ. Geol..

[50] Dołȩgowska S., Migaszewski Z.M. (2013). Anomalous Concentrations of Rare Earth Elements in the Moss-Soil System from South-Central Poland. Environ. Pollut..

[51] Fedotov P.S., Rogova O.B., Dzhenloda R.K., Karandashev V.K. (2019). Metal-organic complexes as a major sink for rare earth elements in soils. Environ. Chem..

[52] Ding S., Liang T., Zhang C., Yan J., Zhang Z., Sun Q. (2005). Role of Ligands in Accumulation and Fractionation of Rare Earth Elements in Plants: Examples of Phosphate and Citrate. Biol. Trace Elem. Res..

[53] Sanchez M.J., Sirvent C.P., Desarro C.D., Sosten Y. (2007). Niveles de fondo y niveles genéricos de referencia de metales pesados en suelos de la Región de Murcia. Ord. Del. Territ..

[54] Instituto Geológico y Minero de España (2007). Determinación de Niveles de Fondo y Niveles Genéricos de Referencia para Metales en Suelos de la Comunidad Autónoma de Aragon.

[55] Sahoo P.K., Powell M.A., Martins G.C., Dall’Agnol R., Salomão G.N., Mittal S., Pontes P.R.M., Guimarães J.T.F., de Siqueira J.O. (2021). Occurrence, distribution, and environmental risk assessment of heavy metals in the vicinity of Fe-ore mines: A global overview. Toxin Rev..

[56] Ballesta R.J., Bueno P.C., Rubi J.A.M., Gim’enez R.G. (2010). Pedo-geochemical baseline content levels and soil quality reference values of trace elements in soils from the Mediterranean (Castilla La Mancha, Spain). Cent. Eur. J. Geosci..

[57] Galhardi J.A., Leles B.P., de Mello J.W.V., Wilkinson K.J. (2020). Bioavailability of trace metals and rare earth elements (REE) from the tropical soils of a coal mining area. Sci. Total Environ..

[58] Li G., Lu N., Wei Y., Zhu D. (2018). Relationship between Heavy Metal Content in Polluted Soil and Soil Organic Matter and pH in Mining Areas. IOP Conf. Ser. Mater. Sci. Eng..

[59] Zhou S., Hursthouse A., Chen T. (2019). Pollution Characteristics of Sb, As, Hg, Pb, Cd, and Zn in Soils from Different Zones of Xikuangshan Antimony Mine. J. Anal. Methods Chem.

[60] Burger A., Lichtscheidl-Schultz I. (2018). Strontium in the environment: Review about reactions of plants towards stable and radioactive strontium isotopes. Sci. Total Environ..

[61] Adriano D.C. (2001). Trace Elements in Terrestrial Environments.

[62] Moreno-Jimenez E., Peñalosa J.M., Manzano R., Carpena-Ruiz R.O., Gamarra R., Esteban E. (2009). Heavy metals distribution in soils surrounding an abandoned mine in NW Madrid (Spain) and their transference to wild flora. J. Hazard. Mater..

[63] Loell M., Albrecht C., Felix-Henningsen P. (2011). Rare earth elements and relation between their potential bioavailability and soil properties, Nidda catchment (Central Germany). Plant Soil.

[64] Oyarzun R., Lillo J., López-García J.A., Esbrí J.M., Cubas P., Llanos W., Higueras P. (2011). The Mazarrón Pb–(Ag)–Zn mining district (SE Spain) as a source of heavy metal contamination in a semiarid realm: Geochemical data from mine wastes, soils, and stream sediments. J. Geochem. Explor..

[65] Martínez-Martínez S., Acosta J.A., Faz Cano A., Carmona D.M., Zornoza R., Cerda C. (2013). Assessment of the lead and zinc contents in natural soils and tailing ponds from the Cartagena-La Unión mining district, SE Spain. J. Geochem. Explor..

[66] Alvarez-Rogel J.e., Penalver-Alcala’ A., Jim’enez-Carceles’ F.J., Tercero M.C., Gonzalez-Alcaraz M.N. (2021). Evidence supporting the value of spontaneous vegetation for phytomanagement of soil ecosystem functions in abandoned metal(loid) mine tailings. Catena.

[67] Kabata-Pendias A. (2010). Trace Elements in Soils and Plants.

[68] Ha N.T.H., Ha N.T., Nga T.T.H., Minh N.N., Anh B.T.K., Hang N.T.A., Duc N.A., Nhuan M.T., Kim K.-W. (2019). Uptake of arsenic and heavy metals by native plants growing near Nui Phao multi-metal mine, northern Vietnam. Appl. Geochem..

[69] Mahdavian K., Ghaderian S.M., Torkzadeh-Mahani M. (2017). Accumulation and phytoremediation of Pb, Zn, and Ag by plants growing on Koshk lead–zinc mining area, Iran. J. Soils Sediments.

[70] Hasnaoui S.E., Fahr M., Keller C., Levard C., Angeletti B., Chaurand P., Triqui Z.E.A., Guedira A., Rhazi L., Colin F. (2020). Screening of Native Plants Growing on a Pb/Zn Mining Area in Eastern Morocco: Perspectives for Phytoremediation. Plants.

[71] Sinha V., Pakshirajan K., Chaturvedi R. (2018). Chromium tolerance, bioaccumulation and localization in plants: An overview. J. Environ. Manag..

[72] Wiche O., Heilmeier H. (2016). Germanium (Ge) and rare earth element (REE) accumulation in selected energy crops cultivated on two different soils. Miner. Eng..

[73] Wen-Shen L. (2020). Phytoextraction of rare earth elements from ion-adsorption mine tailings by Phytolacca americana: Effects of organic material and biochar amendment. J. Clean. Prod..

[74] Saatz J., Vetterlein D., Mattusch J., Otto M., Daus B. (2015). The influence of gadolinium and yttrium on biomass production and nutrient balance of maize plants. Environ. Pollut..

[75] Dinh T., Dobo Z., Kovacs H. (2022). Phytomining of rare earth elements—A review. Chemosphere.

[76] Wang X., Liu D.W. (2017). Integration of cerium chemical forms and subcellular distribution to understand cerium tolerance mechanism in the rice seedlings. Environ. Sci. Pollut. Control. Ser..

[77] Ding S., Liang T., Zhang C., Yan J., Zhang Z. (2005). Accumulation and fractionation of rare earth elements (REEs) in wheat: Controlled by phosphate precipitation, cell wall absorption and solution complexation. J. Exp. Bot..

[78] Staff S.S.D. (1993). Soil Survey Manual.

[79] Beretta A.N., Silbermann A.V., Paladino L., Torres D., Bassahun D., Musselli R., García-Lamohte A. (2014). Soil texture analyses using a hydrometer: Modification of the Bouyoucos method. Cienc. Investig. Agrar..

[80] Chapman H.D. (1965). Cation-Exchange Capacity. Methods of Soil Analysis.

[81] Risser J.A., Baker D.E. (1990). Testing Soils for Toxic Metals. Soil Testing and Plant Analysis.

[82] Crock J.G., Severson R.C. (1980). Four Reference Soil and Rock Samples for Measuring Element Availability in the Western Energy Regions.

[83] Terán-Mita T.A., Faz A., Salvador F., Arocena J.M., Acosta J.A. (2013). High altitude artisanal small-scale gold mines are hot spots for Mercury in soils and plants. Environ. Pollut..

[84] Moreno-Barriga F., Faz Á., Acosta J.A., Soriano-Disla M., Martínez-Martínez S., Zornoza R. (2017). Use of Piptatherum miliaceum for the phytomanagement of biochar amended Technosols derived from pyritic tailings to enhance soil aggregation and reduce metal(loid) mobility. Geoderma.

[85] Acosta J.A., Abbaspour A., Martínez G.R., Martínez-Martínez S., Zornoza R., Gabarrón M., Faz A. (2018). Phytoremediation of mine tailings with Atriplex halimus and organic/inorganic amendments: A five-year field case study. Chemosphere.

[86] Hakanson L. (1980). An ecological risk index for aquatic pollution control.a sedimentological approach. Water Res..

[87] Martínez-Martínez S.M. (2009). Niveles de Fondo y de Referencia de Metales Pesados en Suelos Desarrollados de Material Parental Volcánico, Metamórfico y Sedimentario en la Región de Murcia Espana.

