# Supplementary material for: Phytoremediation Potential of Native Plant Species in Mine Soils Polluted by Metal(loid)s and Rare Earth Elements
Source: Plants (Basel). 2023 Mar 7;12(6):1219. doi: 10.3390/plants12061219 (PMC10058974; doi:10.3390/plants12061219)
Supplement: Supplementary file 1 [file plants-12-01219-s001.zip › plants-2243498-supplementary.pdf]

Table S1- Linear correlation matrix of pH, EC, sand, silt, clay and metal(loid)s/REEs in all soil samples (n = 20).

	pH	EC	Sand	Silt	Clay		pH	EC	Sand	Silt	Clay
V-Tot	0.69**	-0.55*	0.50*	-0.03	-0.43	Bi-Tot	0.00	0.15	0.49*	0.48*	-0.62**
V-Avai	0.47*	0.47*	-0.09	0.60**	-0.15	Bi-Avai	-0.07	0.04	0.30	0.59**	-0.49*
V-Sol	0.60**	0.45*	-0.21	0.33	0.06	Bi-Sol	nd	nd	nd	nd	nd
Mn-Tot	0.00	0.24	0.31	0.67**	-0.53*	Th-Tot	-0.50*	-0.54*	0.18	-0.24	-0.07
Mn-Avai	-0.27	0.12	0.24	0.48*	-0.39	Th-Avai	-0.04	0.05	-0.15	0.06	0.11
Mn-Sol	-0.52*	-0.25	0.54*	0.15	-0.53*	Th-Sol	-0.12	0.10	0.17	0.22	-0.23
Co-Tot	0.34	0.31	0.10	0.48*	-0.27	Ge-Tot	-0.72**	-0.61**	0.56**	-0.12	-0.45*
Co-Avai	0.09	0.22	-0.22	0.41	0.44	Ge-Avai	0.41	0.32	0.04	0.13	-0.008
Co-Sol	-0.49*	0.07	0.60**	0.18	-0.60**	Ge-Sol	-0.03	0.18	0.58**	0.55*	-0.72**
Ni-Tot	-0.14	0.13	0.59**	0.11	-0.56**	Rb-Tot	-0.08	-0.10	-0.31	-0.18	0.34
Ni-Avai	0.004	-0.04	-0.09	0.21	-0.002	Rb-Avai	-0.09	0.36	0.40	0.57**	-0.57**
Ni-Sol	-0.54*	-0.16	0.47*	0.1	-0.45*	Rb-Sol	-0.08	0.34	0.35	0.57**	-0.53*
Cu-Tot	0.05	0.26	0.44*	0.62**	-0.63**	Cs-Tot	0.45*	0.30	-0.58**	0.02	-0.50*
Cu-Avai	-0.05	0.24	0.46*	0.67**	-0.66**	Cs-Avai	nd	nd	nd	nd	nd
Cu-Sol	-0.41	0.03	0.67**	0.28	-0.70**	Cs-Sol	0.2	0.32	-0.15	0.34	0.006
As-Tot	0.06	0.01	0.23	0.20	-0.28	U-Tot	-0.73**	-0.30	0.53*	-0.09	-0.43
As-Avai	0.23	0.51*	0.07	0.68**	-0.32	U-Avai	-0.05	0.44	0.38	0.11	-0.38
As-Sol	0.38	0.41	-0.006	0.49*	-0.18	U-Sol	-0.18	0.28	0.24	0.15	-0.27
Sr-Tot	0.54*	0.56**	-0.08	0.45*	-0.09	Pb-Tot	-0.13	0.22	0.42	0.29	-0.48*
Sr-Avai	0.39	0.32	-0.39	0.12	0.30	Pb-Avai	-0.29	0.01	0.42	0.19	-0.45
Sr-Sol	0.18	0.67**	0.17	0.42	-0.32	Pb-Sol	-0.33	0.08	0.60**	0.53*	-0.73**
Y-Tot	-0.53*	-0.49*	0.46*	-0.02	-0.39	Zn-Tot	-0.85**	-0.46*	0.53*	-0.06	-0.44*
Y-Avai	-0.78**	-0.41	0.40	-0.15	-0.29	Zn-Avai	-0.29	0.08	0.36	0.52*	-0.52*
Y-Sol	-0.51*	-0.29	0.54*	0.24	-0.57**	Zn-Sol	-0.50*	-0.21	0.52*	.0.29	-0.57**
Zr-Tot	0.57**	0.34	-0.44*	0.16	0.33	Al-Tot	-0.62**	-0.63**	0.25	-0.43	-0.05
Zr-Avai	0.20	-0.06	-0.32	-0.02	0.29	Al-Avai	-0.36	-0.38	0.43	0.10	-0.42
Zr-Sol	-0.24	-0.21	0.24	0.12	-0.26	Al-Sol	0.04	-0.37	0.11	0.21	-0.18
Mo-Tot	-0.45*	-0.26	0.83**	-0.05	-0.71**	Fe-Tot	-0.80**	-0.41	0.68**	-0.10	-0.56*
Mo-Avai	-0.016	0.24	0.30	0.69**	-0.53*	Fe-Avai	-0.46*	-0.05	0.29	0.28	-0.36
Mo-Sol	0.42	0.27	-0.26	0.24	0.14	Fe-Sol	-0.21	-0.2	0.50*	0.25	-0.54*
Cd-Tot	-0.46*	-0.14	0.54*	-0.10	-0.44	Cr-Tot	-0.13	-0.13	0.48*	-0.27	-0.31
Cd-Avai	-0.75**	-0.15	0.69**	0.17	-0.68**	Cr-Avai	0.30	0.64**	-0.36	0.17	0.25
Cd-Sol	-0.46*	-0.05	0.63**	0.35	-0.69**	Cr-Sol	-0.41	-0.28	0.51*	0.02	-0.46*
In-Tot	-0.36	-0.06	0.56**	0.04	-0.51*	Se-Tot	-0.29	-0.33	0.60**	0.01	-0.54
In-Avai	0.09	-0.12	0.12	-0.21	0.02	Se-Avai	-0.48*	-0.10	0.32	-0.01	-0.28
In-Sol	-0.22	-0.06	0.47*	0.04	-0.44	Se-Sol	0.41	0.25	0.28	0.36	-0.39
La-Tot	-0.58**	-0.57**	0.31	-0.24	-0.18	Sn-Tot	-0.62**	-0.39	0.63**	0.24	-0.65**
La-Avai	-0.51*	-0.01	0.42	0.35	-0.50*	Sn-Avai	nd	nd	nd	nd	nd
La-Sol	0.04	0.19	0.28	0.48*	-0.44	Sn-Sol	nd	nd	nd	nd	nd
Ce-Tot	-0.57**	-0.58**	0.28	-0.25	-0.15	Sb-Tot	0.05	0.09	-0.13	-0.17	0.18
Ce-Avai	-0.19	0.13	0.15	0.39	-0.28	Sb-Avai	0.26	0.60**	-0.16	0.65**	-0.10
Ce-Sol	0.001	.15	0.31	0.49*	-0.46*	Sb-Sol	0.59**	0.71**	-0.44	0.35	0.25
Pr-Tot	-0.61**	-0.58**	0.29	0.26	-0.16						
Pr-Avai	-0.49*	-0.05	0.27	0.18	-0.30						
Pr-Sol	-0.14	0.07	0.36	0.46*	-0.49*						

Tot : total, Avai: available Sol: water soluble ,**Correlation is significant at the 0.01 level , *Correlation is significant at the 0.05 level (2-tailed), nd: non detectable

Table S2- Linear correlation matrix of total metal(loid)s and REEs in all soil samples (n = 20).

	V	Mn	Co	Ni	Cu	As	Sr	Y	Zr	Mo	Cd	In	La	Ce	Pr	Bi	Th	Ge	Rb	Cs	U	Pb	Zn	Al	Fe	Cr	Se	Sn
V																												
Mn	ns																											
Co	ns	0.78**																										
Ni	ns	ns	0.52*																									
Cu	ns	0.92**	0.86**	0.55*																								
As	ns	0.51*	ns	ns	ns																							
Sr	-0.59**	0.55*	0.81**	ns	0.65**	ns																						
Y	0.54*	ns	ns	ns	ns	ns	-0.51*																					
Zr	ns	ns	0.68**	ns	ns	ns	0.67**	-0.51*																				
Mo	ns	ns	ns	0.6**	ns	ns	ns	0.59**	-0.56**																			
Cd	ns	ns	ns	ns	ns	ns	-0.51*	0.67**	-0.65**	0.64**																		
In	ns	ns	ns	ns	ns	ns	ns	0.49**	ns	0.55*	0.89**																	
La	0.64**	-0.46*	-0.62**	ns	-0.49*	ns	-0.77**	0.73**	-0.67**	0.47*	0.50*	ns																
Ce	0.65**	-0.45*	-0.63**	ns	-0.50*	ns	-0.78**	0.72**	-0.65**	0.44*	0.51*	ns	0.99**															
Pr	0.66**	-0.46*	-0.66**	ns	-0.51*	ns	-0.82**	0.71**	-0.68**	0.44*	0.523*	ns	0.99**	0.99**														
Bi	ns	0.84**	0.61**	ns	-0.86**	0.6**	ns	ns	ns	ns	ns	ns	ns	ns	ns													
Th	0.60**	-0.48*	-0.65**	ns	-0.55*	ns	-0.76**	0.64**	-0.61**	ns	0.44*	ns	0.98**	0.98**	0.98**	ns												
Ge	0.73**	ns	-0.51*	ns	-0.24	ns	-0.76**	0.75**	-0.79**	0.69**	0.67**	0.49*	0.88**	0.88**	0.89**	ns	0.81**											
Rb	0.44*	ns	ns	ns	ns	ns	ns	ns	0.47*	ns	ns	ns	ns	ns	ns	ns	ns	ns										
Cs	ns	ns	ns	ns	ns	ns	ns	-0.56**	0.86**	-0.67**	-0.49*	ns	-0.55*	-0.52*	-0.53*	ns	-0.45*	-0.66**	0.74**									
U	0.51*	ns	-0.59**	ns	ns	ns	-0.68**	0.75**	-0.80**	0.52*	0.78**	0.65**	0.70**	0.69**	0.71**	ns	0.629**	0.796**	ns	-0.69**								
ns	ns	0.58*	ns	ns	0.56*	0.52*	0.09	ns	ns	ns	0.58**	0.83**	ns	ns	ns	0.63**	ns	ns	ns	ns	ns							
Zn	0.68**	-0.16	-0.53*	ns	ns	ns	-0.76**	0.61**	-0.82**	0.52*	0.67**	0.45*	0.72**	0.72**	0.76**	ns	0.65**	0.89**	ns	-0.62**	0.82**	ns						
Al	0.70**	-0.55*	-0.72**	ns	-0.57**	ns	-0.87**	0.59**	-0.62**	ns	0.49*	0.32	0.90**	0.91**	0.93**	ns	0.89**	0.85**	ns	ns	0.67**	ns	0.74**					
Fe	0.74**	ns	ns	ns	ns	ns	-0.70**	0.61**	-0.70**	0.64**	0.70**	0.67**	0.61**	0.60**	0.64**	ns	0.50*	0.85**	ns	-0.54*	0.83**	ns	0.90**	0.69**				
Cr	ns	ns	ns	0.53*	ns	ns	ns	0.45**	ns	0.55*	0.67**	0.74**	ns	ns	ns	ns	ns	ns	ns	ns	ns	0.56*	ns	ns	ns			
Se	ns	ns	ns	0.52*	ns	ns	ns	0.73**	-0.47*	0.78**	0.76**	0.70**	0.53*	0.52*	0.50*	ns	0.46*	0.65**	ns	-0.53*	0.57**	ns	ns	ns	0.49*	0.74*		
Sn	0.68**	ns	ns	0.45*	ns	ns	ns	0.73**	ns	0.66**	ns	ns	0.57**	0.55*	0.55*	ns	0.46*	0.67**	ns	ns	0.50*	ns	0.56**	0.48*	0.62**	ns	0.57*	
Sb	ns	ns	ns	ns	ns	ns	ns	ns	ns	ns	ns	ns	ns	ns	ns	ns	ns	ns	0.46*	ns	ns	ns	ns	ns	ns	ns	ns	ns

**Correlation is significant at the 0.01 level, *Correlation is significant at the 0.05 level (2-tailed), Ns non significant
